# Mitophagy-promoting agents and their ability to promote healthy-aging

**DOI:** 10.1042/BST20221363

**Published:** 2023-09-01

**Authors:** Vijigisha Srivastava, Einav Gross

**Affiliations:** Faculty of Medicine, IMRIC Department of Biochemistry and Molecular Biology, The Hebrew University of Jerusalem, PO Box 12271, Jerusalem, Israel

**Keywords:** aging, lifespan, mitochondria, mitochondrial autophagy, mitochondrial biogenesis, mitophagy

## Abstract

The removal of damaged mitochondrial components through a process called mitochondrial autophagy (mitophagy) is essential for the proper function of the mitochondrial network. Hence, mitophagy is vital for the health of all aerobic animals, including humans. Unfortunately, mitophagy declines with age. Many age-associated diseases, including Alzheimer's and Parkinson's, are characterized by the accumulation of damaged mitochondria and oxidative damage. Therefore, activating the mitophagy process with small molecules is an emerging strategy for treating multiple aging diseases. Recent studies have identified natural and synthetic compounds that promote mitophagy and lifespan. This article aims to summarize the existing knowledge about these substances. For readers’ convenience, the knowledge is presented in a table that indicates the chemical data of each substance and its effect on lifespan. The impact on healthspan and the molecular mechanism is reported if known. The article explores the potential of utilizing a combination of mitophagy-inducing drugs within a therapeutic framework and addresses the associated challenges of this strategy. Finally, we discuss the process that balances mitophagy, i.e. mitochondrial biogenesis. In this process, new mitochondrial components are generated to replace the ones cleared by mitophagy. Furthermore, some mitophagy-inducing substances activate biogenesis (e.g. resveratrol and metformin). Finally, we discuss the possibility of combining mitophagy and biogenesis enhancers for future treatment. In conclusion, this article provides an up-to-date source of information about natural and synthetic substances that activate mitophagy and, hopefully, stimulates new hypotheses and studies that promote healthy human aging worldwide.

## Introduction

“Art is the elimination of the unnecessary” (Picasso), and by analogy, so is mitophagy for the mitochondrial network.

Healthy mitochondria are essential for many fundamental processes in aerobic cells, including the transformation of biomolecule chemical energy to adenosine triphosphate (ATP) via oxidative phosphorylation [1], synthesizing heme [2], degrading fatty acids through β oxidation [3], regulating calcium [4] and iron [5] homeostasis, and controlling cell death by apoptosis [6]. Moreover, mitochondria generate reactive oxygen species (ROS) that play crucial functions in various cellular and physiological processes, including redox homeostasis and immunity [7,8]. In contrast, damaged mitochondria lead to ATP depletion [9], impaired metal iron homeostasis [10,11], excessive ROS formation [9], and cell death activation through the release of cytochrome C [9,12]. Therefore, eliminating injured mitochondria by a process called mitochondrial autophagy (mitophagy) is crucial for proper cellular activity and health.

Multiple mitophagy pathways involve different signaling molecules, enzymes, and adaptor proteins. Below, we briefly describe three canonical pathways — excellent in-depth and up-to-date reviews about the different mitophagy pathways [13–15].

### PINK1/PARKIN-dependent mitophagy

**Figure 1.**
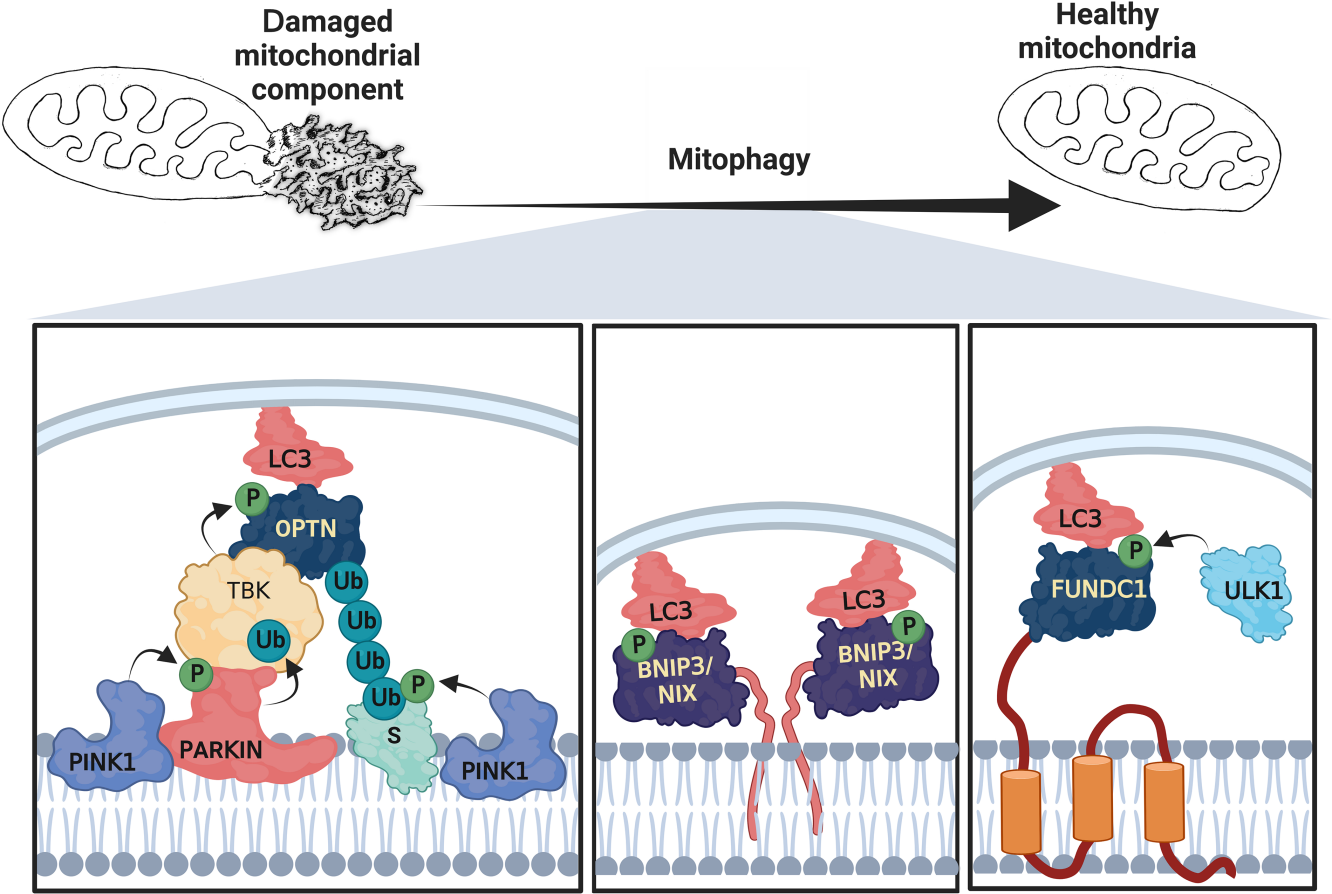
Schematic illustration of three mitophagy pathways (left to right, inspired by [23]). PINK1/Parkin-dependent mitophagy: The decrease in membrane potential in damaged mitochondrial regions leads to the accumulation of PINK1 on the outer mitochondrial membrane (OMM), where it recruits Parkin. PINK1 activates Parkin by phosphorylating it within its ubiquitin-like domain, releasing its activity. Parkin then ubiquitinates OMM substrates (S) such as MFN-2 and Vdac1. These ubiquitinated substrates are bound by cargo receptors such as OPTN1 and NDP52, which interact with membrane-anchored LC3 (LC3B-II) to promote mitophagy. The recruitment of TBK1 to the OMM promotes the binding of OPTN1 and cargo receptors to the ubiquitinated substrates, thus initiating mitophagy. BNIP3/NIX-dependent mitophagy: BNIP3 undergoes phosphorylation on residues S17 and S24, which are adjacent to the essential tryptophan residue at W18. This phosphorylation event promotes the interaction of BNIP3 with LC3B-II. FUNDC1-dependent mitophagy: The phosphorylation of S17 on FUNDC1 by ULK1 promotes its interaction with LC3B-II.

In healthy mitochondria, the PTEN-induced kinase 1 (PINK1) is imported to the inner mitochondrial membrane (IMM) and thus targeted to degradation by the presenilin-associated rhomboid-like protein (PARL) and the cytosolic proteasome. However, when mitochondrial membrane potential (MMP) drops, PINK1 accumulates on the outer mitochondrial membrane (OMM) and becomes activated through autophosphorylation [16,17]. PINK1 phosphorylates ubiquitin and thus recruits the E3 ubiquitin ligase parkin to the OMM [16,18]. Moreover, it can fully activate parkin through direct phosphorylation on serine 65 (in the ubiquitin-like domain), which is further enhanced if parkin binds to phospho-ubiquitin [19]. Parkin ubiquitinates multiple OMM proteins including, voltage-dependent anion channel-1 (VDAC1), mitofusin-2 (MFN2), and TANK-binding kinase 1 (TBK1) [20]. The ubiquitinated OMM proteins interact with cargo receptors, i.e. proteins that can simultaneously interact with autophagosome-associated Atg8/LC3 family proteins and ubiquitin [20], including optineurin (OPTN) and nuclear dot protein 52 (NDP52) [16,18] (Figure 1). Apart from the described signaling pathway, the mitophagy pathway mediated by Pink1/Parkin may involve other mitophagy receptors, such as the Prohibitin 2 IMM protein [21,22].

### FUNDC1-dependent mitophagy

FUN 14 domain-containing 1 (FUNDC1) is an OMM protein conserved from the nematode *Caenorhabditis elegans* (*C. elegans*) to humans [24]. Studies from recent years show that FUNDC1 plays a vital function in hypoxia-induced mitophagy [25]. FUNDC1 has three transmembrane regions and an amino-terminal LC3 interacting region (LIR) that can interact with Atg8/LC3 family proteins (Figure 1, [25]). This interaction is tightly regulated by phosphorylation [26]. When oxygen is not limiting (normoxic conditions), SRC and CK1 phosphorylate FUNDC1 at tyrosine 18 (Y18) and serine 13 (S13), respectively, thus decreasing its affinity to LC3 [26,27]. However, in hypoxia these residues are dephosphorylated. Hence, the interaction with LC3 increases. In contrast, FUNDC1 phosphorylation at serine 17 (S17, adjacent to the LIR sequence motif) by the Unc-51-like autophagy activating kinase 1 (ULK1) significantly increases its interaction with LC3 [26].

### BNIP3/NIX-dependent mitophagy

The BCL2 interacting protein 3 (BNIP3) and BCL2 interacting protein 3 like (NIX) are stress-induced mitophagy receptors originally categorized as proapoptotic BH3 proteins. Like FUNDC1, BNIP3/NIX are mitochondrial transmembrane proteins (with a single transmembrane region) that project their LIR motif into the cytoplasm, where it can directly interact with LC3 and other Atg8/LC3 family proteins [28–30] (Figure 1). This interaction is regulated, in part, by the phosphorylation of serine restudies nearby the LIR motif (serine 17 (S17)) and serine 35 (S35) in BNIP3 and NIX respectively [31,32]). BNIP3/NIX are also regulated at the transcriptional level. One critical transcription factor (TF) that regulates their activity is the Hypoxia Inducible Factor 1 (HIF-1) TF, particularly BNIP3 when O_2_ levels drop below 2% [33–35].

### Other mitophagy pathways

Studies from recent years identified alternative mitophagy pathways. This includes the BCL2-13 and lipid (e.g. cardiolipin and ceramide)-dependent mitophagy pathways [23]. Due to space limitations, we do not describe these pathways in detail.

*Yet, all mitophagy pathways lead to the same endpoint: the degradation of damaged mitochondrial components by lysosomal enzymes. Therefore, to simplify, we refer to all types of defective mitochondrial clearance pathways as ‘mitophagy.’*

## The effect of mitophagy activating substances on life- and healthspan

Mitophagy progressively declines with age [36,37]. As a result, the accumulation of damaged mitochondria contributes to cellular damage that may lead to the development of various disease states, such as Alzheimer's [38] and Parkinson's diseases [39], idiopathic pulmonary fibrosis (IPF) and aging lung [40], cancer [41], and sarcopenia [42,43]. Therefore, mitophagy enhancement by pharmacological intervention is an emerging strategy for treating various age-associated diseases. The main goal of this review is to provide an up-to-date resource, both for new researchers in the mitophagy field and for established ones, regarding the variety of substances that: (1) Stimulate the mitophagy process in cells/organisms/animals; (2) Extend the lifespan and healthspan of organisms/animals.

For the reader's convenience, the above information is organized in a table (Table 1), categorizing substances based on their biological activity. It is important to acknowledge that numerous substances can fall into multiple categories. To ensure clarity and ease of reading Table 1, we allocated each substance into a single category that best describes its predominant mode of action. We indicate each ‘substance's chemical formula, molecular weight (MW), structure, and effect on lifespan. Also, we indicate many ‘substances’ healthspan effects and the genes/proteins that mediate their activity. Finally, we indicate in the table which mitophagy-activating substances are natural.

**Table 1:** Effects of mitophagy activators on life span and health span

Mitophagy enhancer	Chemical structure/molecular formula and weight	Mean lifespan extension^1^	Healthspan effect	Genes/proteins involved in lifespan/healthspan extension
**ANTIOXIDANTS**				
Astaxanthin (AST; carotenoid)	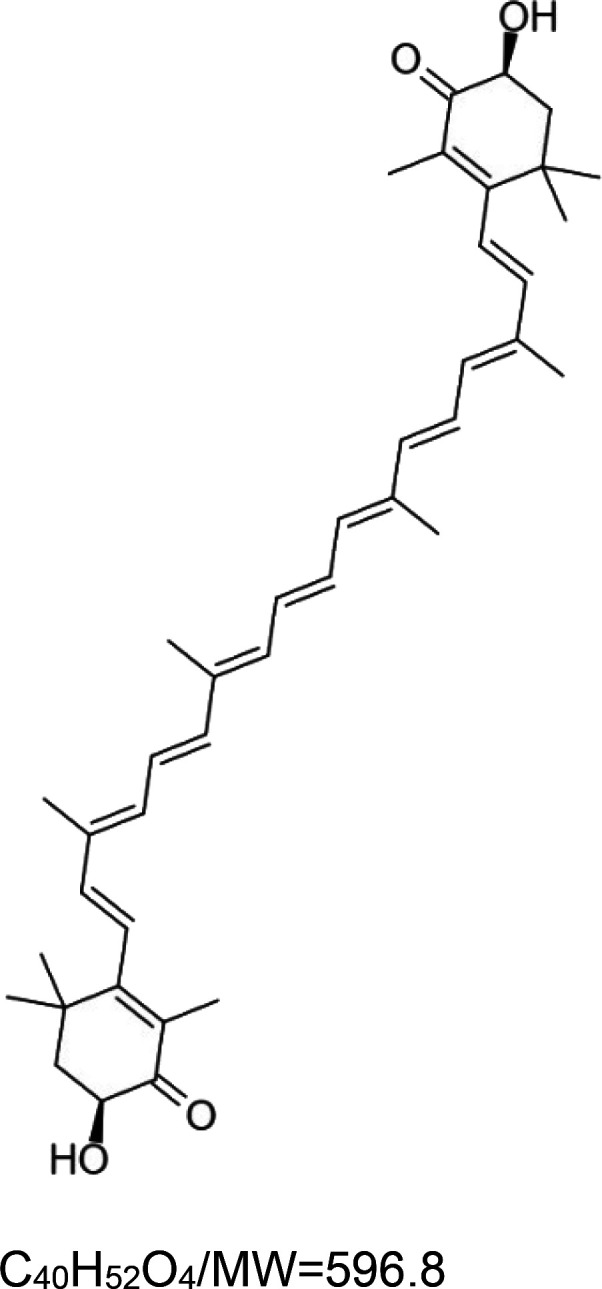	Yeast (*S. cerevisiae*) — 30 µM, 10% extension (after 21 days) [44]. Nematode (*C. elegans*) — 160 µM, 28%, 25%, and 23% extension for the *S*, *R,* and *M* AST isomers, respectively [45]. Moreover, 60, 120, and 240 µM extend lifespan (median) by 9.7%, 13.4% and 5.8%, respectively [46]. Fly (*D. melanogaster*) — 10 and 20 mg/ml of the microalga *Haematococcus pluvialis* (HP), which is a natural producer of astaxanthin, extend the lifespan of SOD^n108^ mutant flies (males) by 15.4% and 34.6%, respectively. However, 20 mg/ml HP significantly decreases the lifespan of wild-type flies [47].	Nematode (*C. elegans*) — 160 µM, AST (S, R, M) increases locomotory activity in aged worms (day 15 of adulthood) [45], and 120 µM AST also increases locomotor activity [46]. Also, 60 µM AST increases locomotory activity in elderly worms (day 11, head swings and body bends). Notably, AST significantly decreases pharyngeal pumping [48]. Fly (*D. melanogaster*) — AST source (HP) rescues the climbing defect of SOD^n108^ flies (males) [47].	
Baicalein (natural polyphenol)	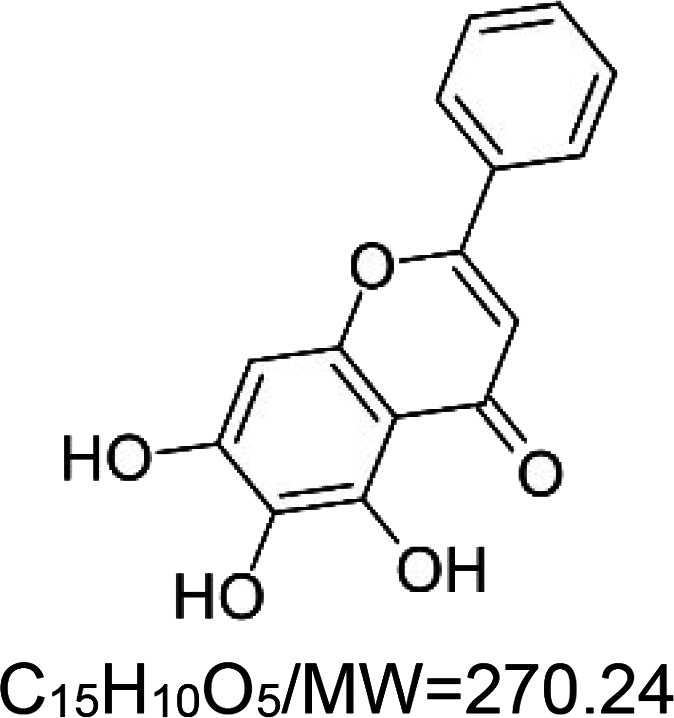	Nematode (*C. elegans*) — 100 µM, 45% extension [49] and 10 µM, 16.5% extension [50]. Also, 0.1% baicalein increases mean lifespan by 36.7% [51]. Fly (*D. melanogaster*) — 0.04, 0.2, 1 mg/ml result in 8.5%, 19.8%, and 9.3% extensions, respectively, in male flies [52].	Fly (*D. melanogaster*) — 0.04, 0.2, 1 mg/ml result in 2.95, 2.92, and 0.71 fold increase in fertility (laid pupae) [52]. Rat (*Rattus norvegicus*) — Neuroprotective function in PD model (induced by the neurotoxin 6-OHDA) [53].	Nematode (*C. elegans*) — *cbp-1* (CBP/p300 homolog) is essential for baicalein activity [51]. Rat (*Rattus norvegicus*) — miR-30b-5p and the SIRT1/AMPK/mTOR pathway [53].
Carnosic acid (natural terpenoids)	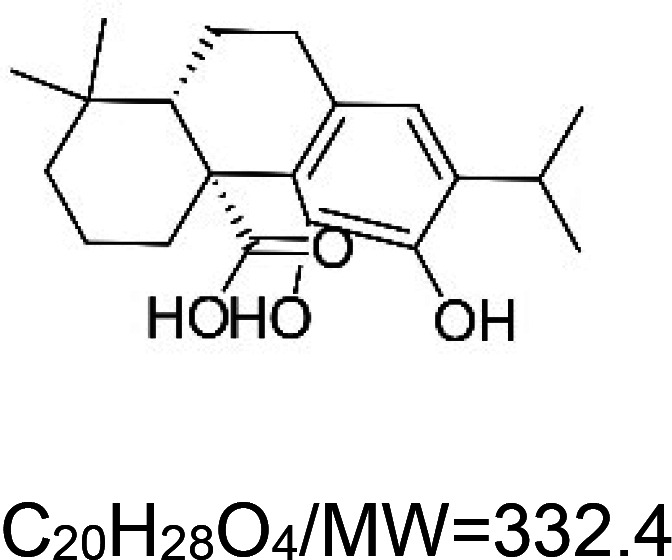	Nematode (*C. elegans*) — 60, 120, and 180 µM result in 3.4%, 8.3%, and 15.8% extension [54].	Nematode (*C. elegans*) — 60, 120, and 180 µM improve locomotory activity in 10 and 14 days worms [54]. Moreover 180 µM ameliorates paralysis induced by amyloid beta and polyglutamine by 18.6% and 12.6%, respectively [54]. Mouse (*M. musculus*) — Carnosic acid (0.13 mg per kg, twice a week, with 2–3 days intervals) improves the motor function of 133 days old mice bearing the human SOD1 G93A mutation [55].	
Carnosol (natural Terpenoids)	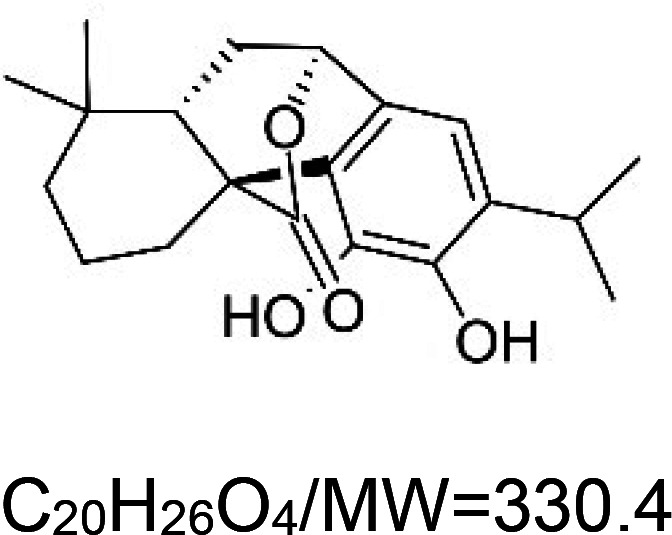	Nematode (*C. elegans*) — 180 µM, 19% extension [56].	Nematode (*C. elegans*) — 180 µM improves locomotory activity (body bends and spontaneous movement). Also, the same concentration ameliorates paralysis induced by amyloid beta and polyglutamine [56].	Nematode (*C. elegans*) — The heat shock transcription factor HSF-1 is essential for the lifespan extension induced by carnosol [56].
Curcumin (natural polyphenol)	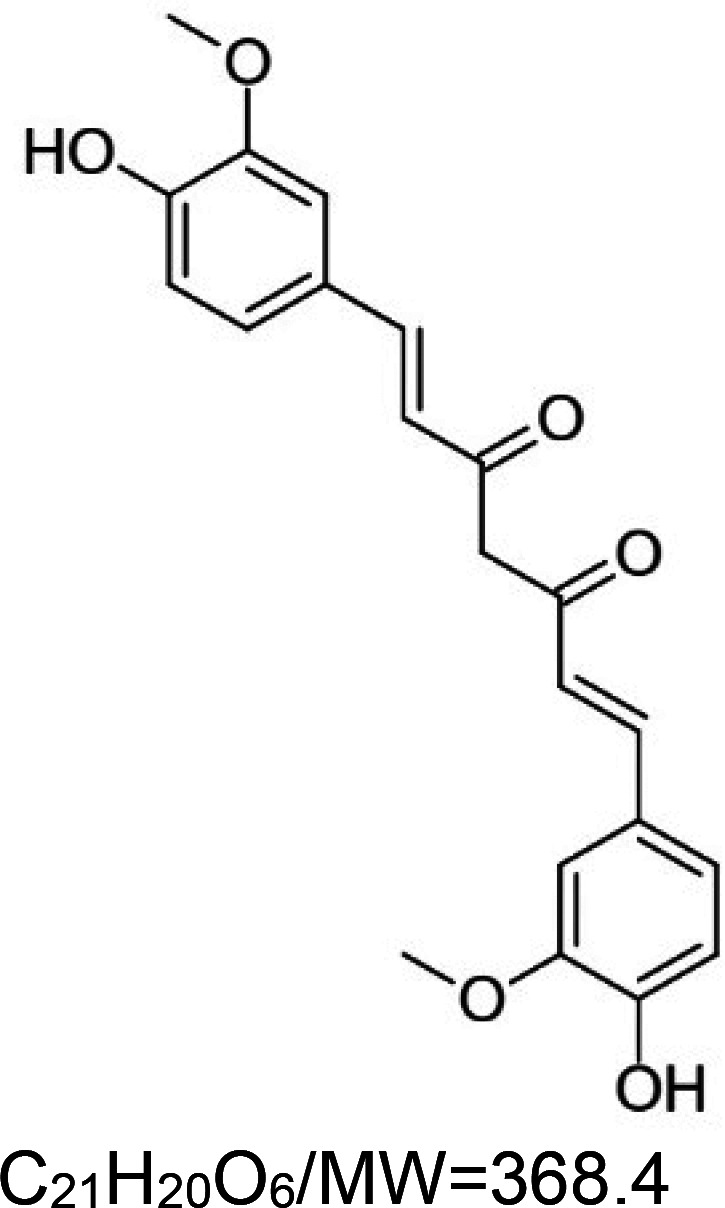	Yeast (*S. cerevisiae*) — 200 and 300 µM, ∼2.8 fold extension [57]. Nematode (*C. elegans*) — 20 µM, 39% extension [58]. Also, 10, 25, 50, and 100 µM result in 10.6%, 20.1%, 13.8%, and 12% extensions, respectively [59]. Fly (*D. melanogaster*) — 100 µM, 19% extension (only in Canton-S females). In contrast, 250 µM extends the lifespan of Ives males (16%) but not females [60]. In addition, 20.3% extension at 1 mg/g of media (males) [61] and 25% extension (median lifespan, when given to the larvae, days −9 to −5) [62]. Mouse (*M. musculus*) — 2000 mg/kg of food, no significant change (males & females) [63].	Fly (*D. melanogaster*) — Enhanced spontaneous movement in elderly females (day 35) but not in elderly males. Increased climbing activity in both females and males (day 35) [60].	Nematode (*C. elegans*) — lifespan extension requires *osr-1*, *sek-1*, *mek-1*, *skn-1*, *unc-43*, *sir-2.1*, and *age-1* [58].
Fisetin (natural polyphenol)	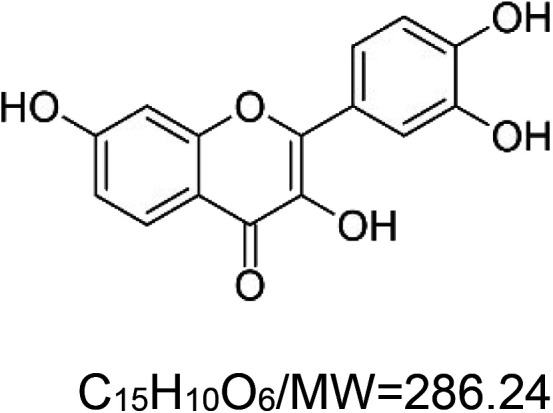	Yeast (*S. cerevisiae*) — 10 µM, 55% extension [64]. Fly (*D. melanogaster*) — 100 µM, 23% extension (males & females) [65]. Mouse (*M. musculus*) — 500 ppm, 11% (males & females) [66].	Mouse (*M. musculus*) — Decreases senescence of T and NK cells [66].	
Hydroxytyrosol (natural phenol)	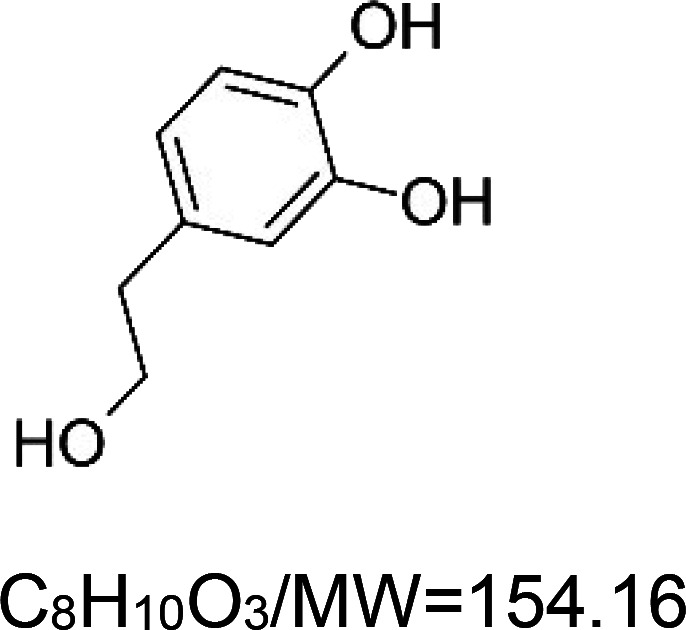	Nematode (*C. elegans*) — 250 µg/ml, 14% extension [67].	Nematode (*C. elegans*) — Hydroxytyrosol enhances the locomotory activity of wild-type worms. Moreover, it decreases the toxic effect of alpha-synuclein in two *C. elegans* Parkinson's disease models, the OW13 and UA44 strains, respectively [67].	
Kaempferol (natural polyphenol)	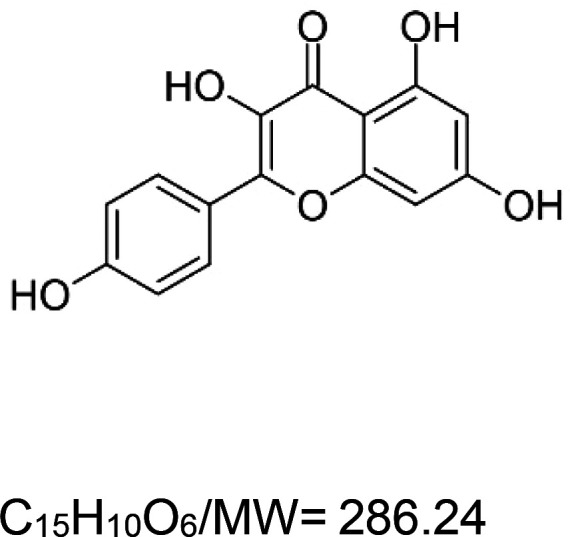	Nematode (*C. elegans*) — 100 µM, 5.6% extension [68].	Mouse (*M. musculus*) — 25 mg/kg body weight per day results in weight-loss of 5–7%, decreased cholesterol and blood-insulin (at 9 & 15, and after 12 months of treatment, respectively), improved rotartod performances (after 12 and 18 months of treatments!) [69]. In addition, kaempferol (30 mg per kg body Weight per day, for 30 days) decrease tumor volume and mass by 65.93% and 49.25%, respectively, in a mice model of human liver cancer xenograft [70].	Nematode (*C. elegans*) — Lifespan extension requires DAF-16 and MEV-1 [68].
Melatonin (natural indoleamine)	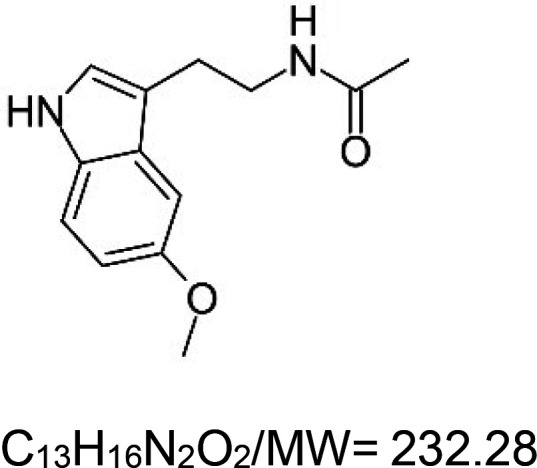	Unicellular ciliates (*P. tetraurelia*) — 10 mg/L, 21–24% extension [71]. Nematode (*C. elegans*) — No effect at 10 mg/L and 100 mg/L. However, higher doses, i.e. 1–100 g/L, decrease lifespan by 31% to ∼57% [72]. Fly (*D. melanogaster*) — 100 µg/ml, 13.5% extension (median life span, males) [73]. Mouse (*M. musculus*) — 20 mg/L, 5.4% extension (in female CBA mice) [74].	Nematode (*C. elegans*) — Melatonin (10 µM) decreases the accumulation of alpha-synuclein in a *C. elegans* Parkinson's disease model strain (NL5901). Moreover, it protects dopaminergic neurons from 6-OHDA-induced damage [75].	
Myricetin (natural polyphenol)	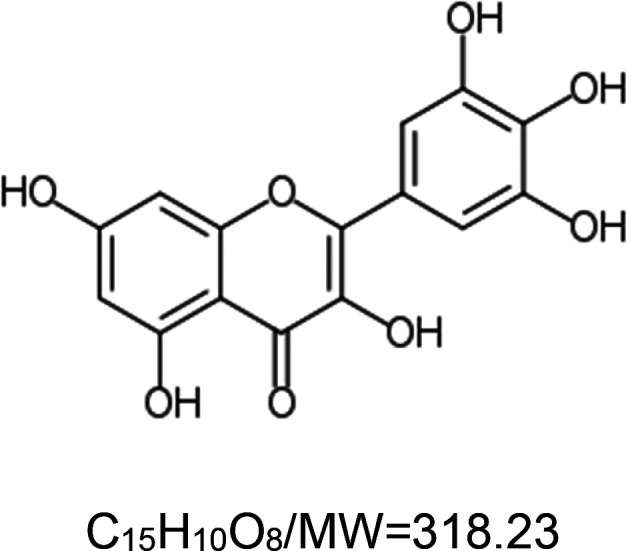	Nematode (*C. elegans*) — 100 µM, 32.9% extension [76] and 18% [68]. Fly (*D. melanogaster*) — 40 µM, 36% extension in a fly model of Parkinson's Disease (PD) [77].	Fly (*D. melanogaster*) — 10, 20 and 40 µM of myricetin postpone climbing ability deterioration by 1.11, 1.26 and 1.40 folds, respectively, in a fly model of PD [77].	Nematode (*C. elegans*) — Lifespan extension requires DAF-16 [76], however, in another study DAF-16 was dispensable [68].
Naringin (natural polyphenol)	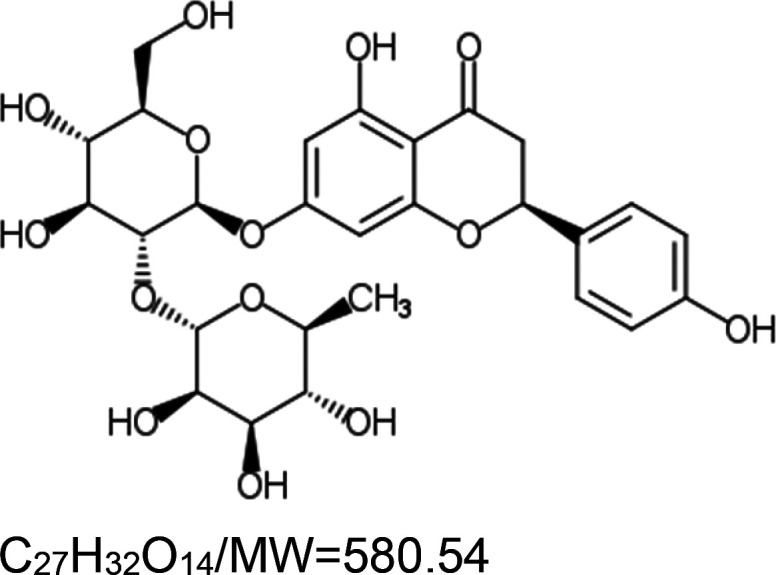	Nematode (*C. elegans*) — 50 µM, 23% extension [78] and 12.7% by 5 µM [79]. Fly (*D. melanogaster*) — No significant effect on females (0.3, 0.5, and 1 µM). However, 1 µM decreases males’ lifespan by 8% [79].	Nematode (*C. elegans*) — 50 µM, improved locomotory activity (body bends) in elderly worms. Also, the same concertation decreases the accumulation of alpha-synuclein in a *C. elegans* Parkinson's disease model strain (NL5901). Moreover, it protects dopaminergic neurons from 6-OHDA-induced damage [78]. Fly (*D. melanogaster*) — Increases fertility (egg laying) in elderly females (3.6 fold), but a negligible effect on the motility of the flies [79].	Nematode (*C. elegans*) — Lifespan extension requires DAF-16 [78] and AAK-2 (AMP-Activated Kinase) [79].
Quercetin (natural polyphenol)	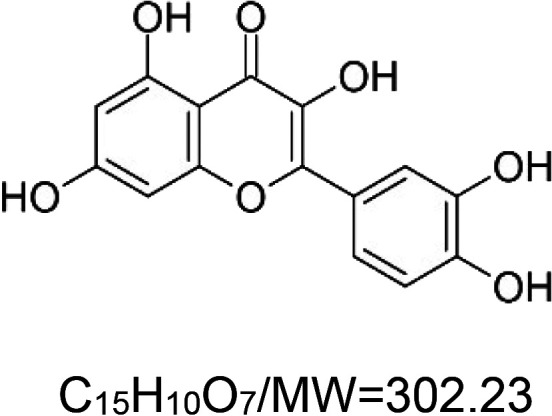	Fungus (*P. anserina*) — 300 µM, 10% extension [80]. Yeast (*S. cerevisiae*) — 0.1 mg/ml, 60% extension [81]. Nematode (*C. elegans*) — 100 µM, 15% extension [82] and 5.8% [68]. Also, 25 µM extends lifespan by 20.9% and by 57% under oxidative stress conditions [83]. Mouse (*M. musculus*) — 0.125 mg/kg body weight shows no significant change in lifespan when given for 17 months (14 to 31) [84].	Nematode (*C. elegans*) — 50 and 500 µM, protect from motility deterioration in elderly worms (9 & 12 days) [85]. Mouse (*M. musculus*) — decreased hair loss, less muscle fibrosis, enhanced diastolic function and exercise performances [84].	Fungus (*P. anserina*) — The O-methyltransferase PaMTH1 is required for the pro-longevity function of quercetin [80]. Nematode (*C. elegans*) — The following genes are required for motility enhancement by quercetin, i.e. *age-1*, *daf-2*, *daf-16*, *nsy-1*, *pmk-1*, *sek-1*, and *skn-1* [85]. MEV-1 is required for lifespan extension, but not DAF-16 [68].
Resveratrol (natural polyphenol)	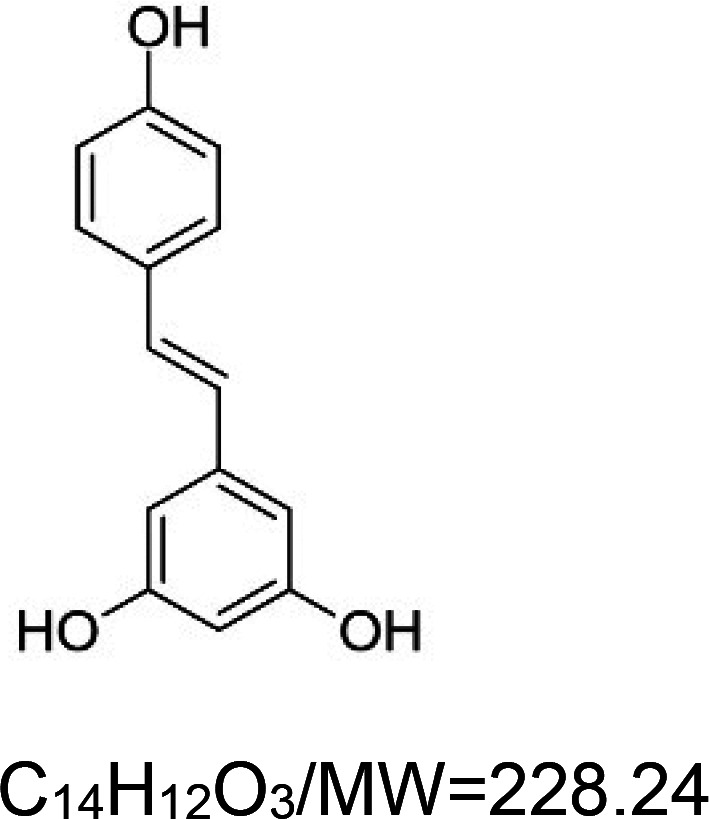	Yeast (*S. cerevisiae*) — 10 µM, 70% extension [64]. Nematode (*C. elegans*) — 100 µg/ml, 11% extension [86]. Nematode (*C. elegans*) — 1000 µM, 18% extension [87]. Fly (*D. melanogaster*) — 200 µM, 10% (males) & 16% (females) extension [88]. In contrast, 500 µM resveratrol does not affect the lifespan of flies (males and females) nor locomotory activity or resistance to oxidative stress [89]. Fish (*N. guentheri*) — 200 µg/g food, 19% extension (males and females) [90] Mouse (*M. musculus*) — both 300 and 1200 ppm, no significant change in both males & females [91].	Nematode (*C. elegans* and *C. briggsae*) — Resveratrol increases swimming in N2 and JU775 worms on days 9 and 12 of adulthood. However, the swimming ability of MY16 worms (another *C. elegans* strain) and *C. briggsae* animals is not improved by resveratrol [92]. Mouse (*M. musculus*) — ameliorates age-related dysfunctions (cataracts, osteoporosis, motor coordination, and vascular function [93].	Nematode (*C. elegans*) — lifespan extension requires the function of *bec-1* [86] and *sir-2.1* [87].
Tomatidine (natural steroidal alkaloid)	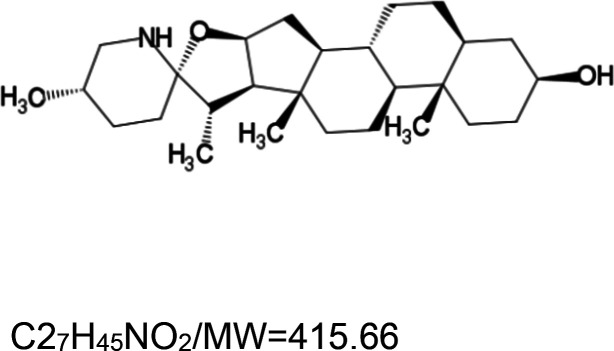	Nematode (*C. elegans*) — 25 µM, 7% extension [94].	Nematode (*C. elegans*) — Tomatidine improves muscle function (i.e. swimming and pharyngeal pumping) in structure in elderly worms [94].	Nematode (*C. elegans*) — The healthspan effect of Tomatidine depends on the activities of the mitophagy proteins DCT-1 and PINK-1 and the transcription factors ATFS-1 and SKN-1 [94].
Urolithin A (natural polyphenol)	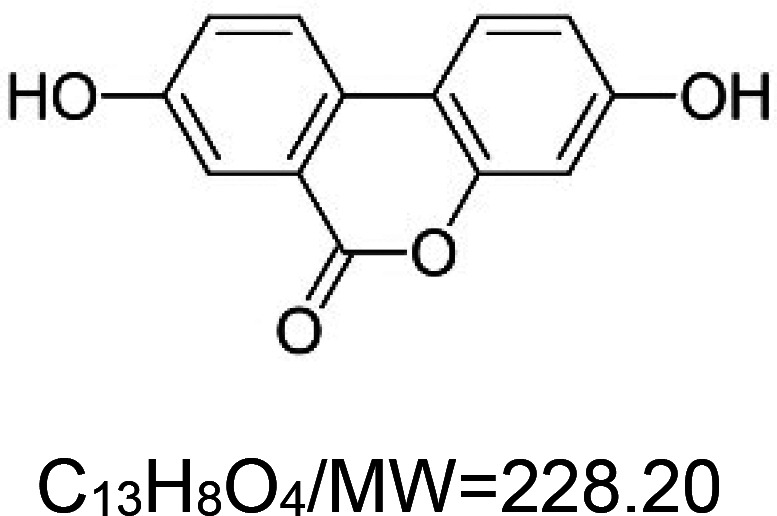	Nematode (*C. elegans*) — 50 µM, 45% extension [95].	Nematode (*C. elegans*) — 50 µM increases pharyngeal pumping, mobility, and muscle-fiber organization in aged worms [95]. Mouse (*M. musculus*) — 2.5 mg per kg body weight (three times a week, for 4 months) results in improved locomotory activity, learning, and memory in an Alzheimer's mouse model [96]. Human (*H. sapiens*) — 500 and 1000 mg of urolithin A (UA) increase muscle strength and aerobic endurance in middle-aged adults (40–64 years) [97].	Partially dependent on AAK-2, and completely dependent on the MEV-1 mitochondrial succinate dehydrogenase complex subunit C [95]. In addition, it requires the following autophagy/mitophagy genes: *bec-1*, *vps-34*, *pink-1*, *dct-1*, *sqst-1*, and *skn-1* [95].
Vanillic acid (phenol)	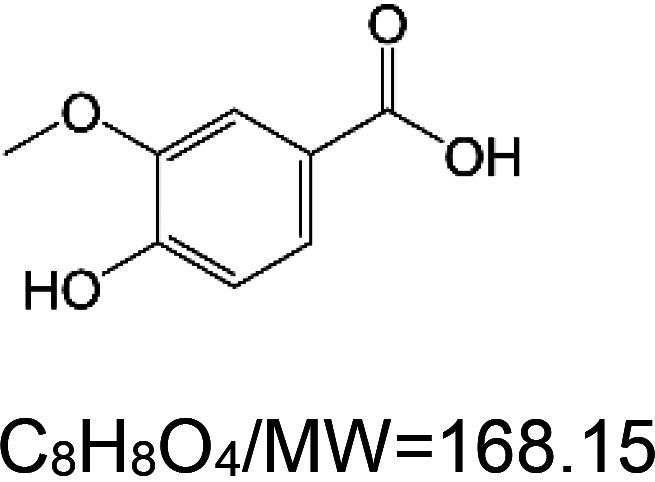	Nematode (*C. elegans*) — 5 mM, 48.8% extension (median survival) [98]. Moreover, 250 µM results in 8.9% extension (median survival) [99].	Nematode (*C. elegans*) — 5 mM vanillic acid ameliorates paralysis induced by amyloid beta and polyglutamine (Q40). Moreover, it preserves the motility of elderly worms (day 12) [98].	Nematode (*C. elegans*) — It appears that vanillic acid function depends on the heat shock factor 1 (HSF-1) protein [98].
**MITOCHONDRIAL UNCOUPLER**				
2,4-dinitrophenol	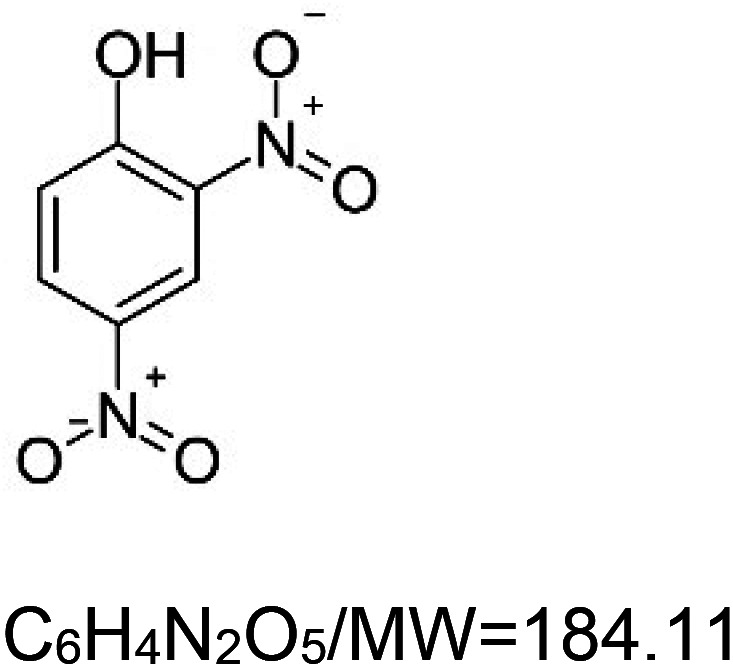	Yeast (*S. cerevisiae*) — 10 nM, 14.7% extension [100]. Nematode (*C. elegans*) — 10 µM does not affect lifespan, however, enhance learning ability (by 20%) and learning ability (by 33%) [101]. Fly (*D. melanogaster*) — 0.1% w/v, 12% extension [102], and 0.8% results in 20% extension [103]. Zebra finch (*Taeniopygia guttata castanotis*) — ∼4 mg per kg per day, 27% decrease in median lifespan [104]. Mouse (*M. musculus*) — 1 mg/L (30-105 µg per kg per day), 7% extension [105].	Mouse (*M. musculus*) — 0.5 mg/kg (once a day, orally) improved short-term memory in an Alzheimer's mouse model (APP/PS1 double mutant mice) [106]. Also, DNP protects against motor dysfunction (1 or 5 mg/kg) and dopaminergic neuronal injury (5 mg/kg) induced by MPTP (a Parkinson's disease model in mice) [107].	
carbonylcyanide-3-chlorophenylhydrazone (CCCP)	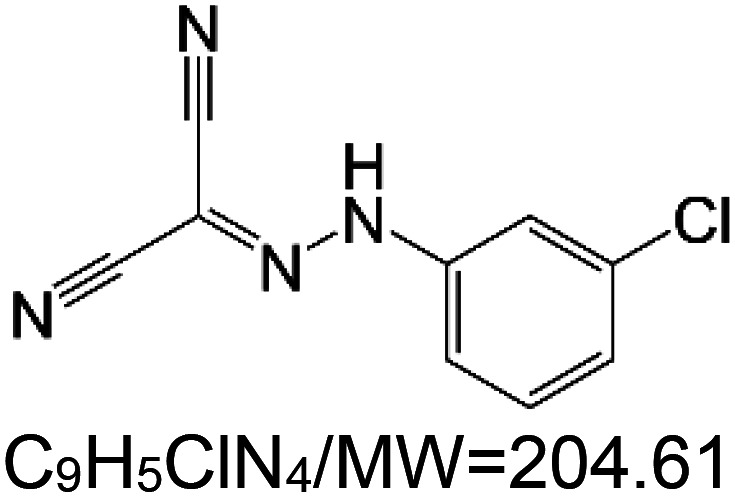	Nematode (*C. elegans*) — 10 and 15 µM, 60% extension in median lifespan [108].		
**MITOCHONDRIAL COMPLEX I INHIBITOR**				
Rotenone (natural insecticide)	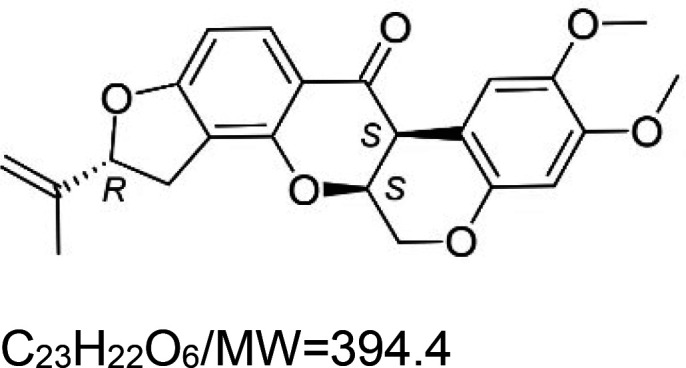	Nematode (*C. elegans*) — 100 nM, 10% extension [109].	Nematode (*C. elegans*) — 100 nM increases locomotion after 7 days of exposure [109].	Nematode (*C. elegans*) — PMK-1 and SKN-1 dependent [109].
**REDOX MODULATORS**				
Polydatin (piceid) (natural polyphenol)	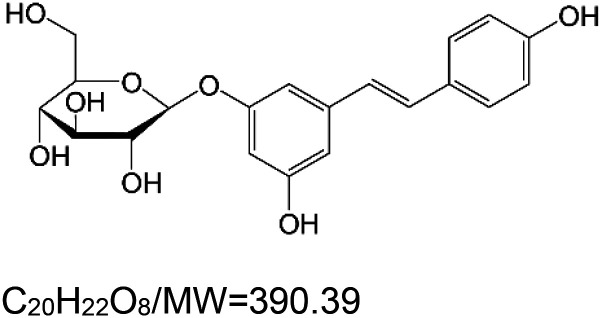	Nematode (*C. elegans*) — 1 mM, 30.7% extension [110].	Nematode (*C. elegans*) — Worms’ mobility (stroke frequency) was significantly improved by 1 mM polydatin [110].	Nematode (*C. elegans*) — Polydatin activity appears to require DAF-16 [110].
**METABOLIC MODULATORS**				
Aspirin ((acetylsalicylic acid; non-steroidal anti-inflammatory drug))	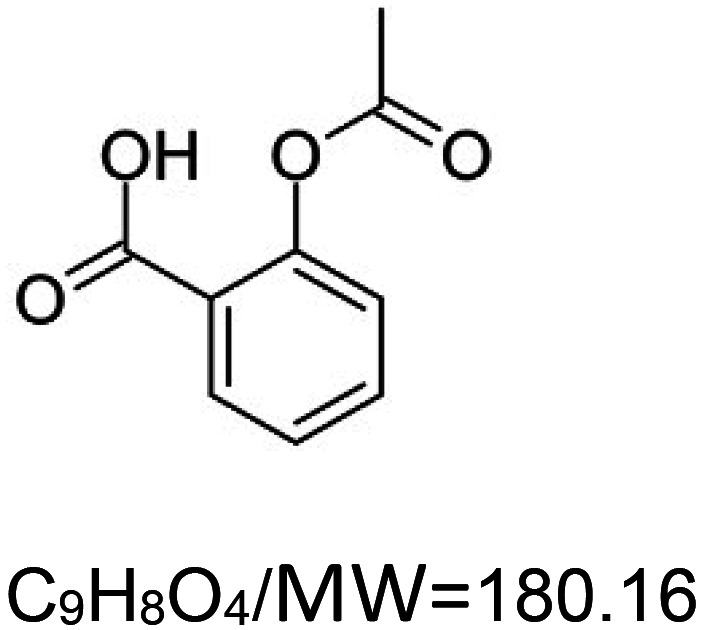	Nematode (*C. elegans*) — 100 µM, 15.5% extension [111]. Fly (*D. melanogaster*) — 0.5 µM (in dietary restriction food, 1xSYA), 12.5% extension [112]. House cricket (*A. domesticus*) — Low dose of aspirin (0.556 mg per gram of food) results in 52% and 67% extension for females and males, respectively. High dose of aspirin (2.263 mg per gram of food) results in 49% and 77% extension for females and males, respectively [113]. Mouse (*M. musculus*) — 21 mg/kg of food, 8% extension (just in males) [114].	Nematode (*C. elegans*) — 100 µM, delayed decline of fast-movement in old animals [111].	DAF-16 (FOXO), AAK-2 (AMP-Activated Kinase) [111].
Dichloroacetate	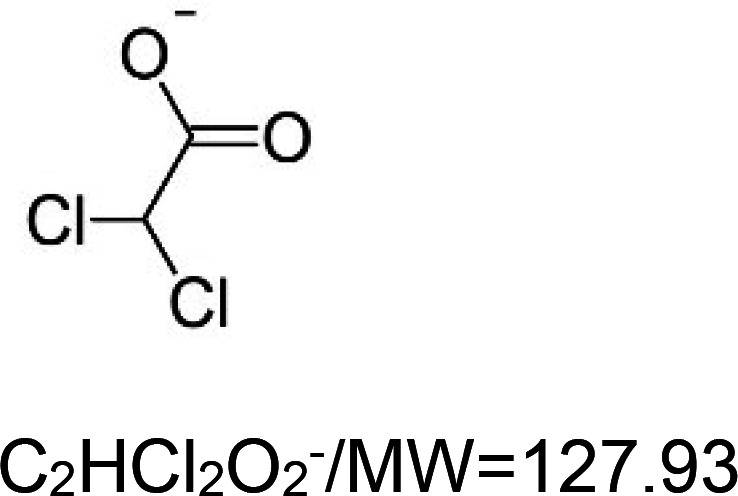	Nematode (*C. elegans*) — 50 µg, ∼7.8% extension [115]. Also, 25 mM dichloroacetate extended the median lifespan of animals treated with *dld-1* RNAi (diluted 20 fold) by ∼32% [116]. Fly (*D. melanogaster*) — 0.5 mg/ml, ∼67% extension in a fly model for sepsis [117]. Moreover, 0.02 mg/ml results in ∼15% extension (male files) [118].	Nematode (*C. elegans*) — 50 µg increases locomotory activity in aged worms [115]. Fly (*D. melanogaster*) — 0.02 mg/ml improved locomotory activity in elderly male flies [118].	
Metformin	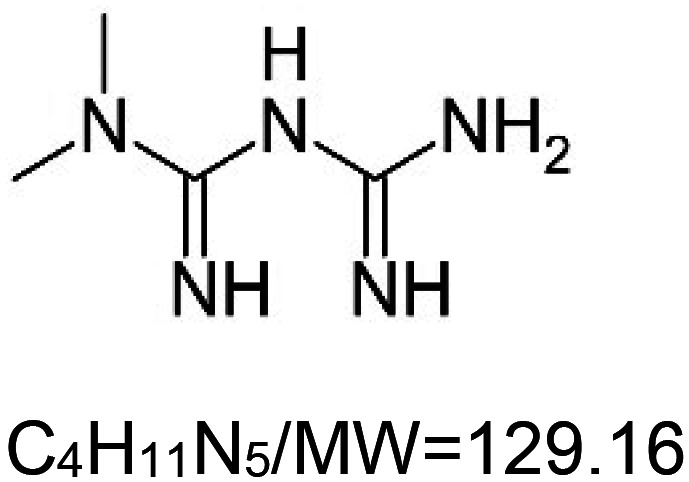	^4^Nematode (*C. elegans*) — 50 mM, 31% [119] and 42% extension [120]. However, when given from day 10 of adulthood, metformin (10, 25, and 50 mM) decreases lifespan (27%) [121]. Fly (*D. melanogaster*) — 5 mM, 17% extension (median, males) [122]. House cricket (*A. domesticus*) — metformin (17.8 mg per gram of food) results in 47% and 39% extension for females and males, respectively [113]. Killifish (*N. Guentheri*) — 2 mg per gram food, 34.7% extension (just in male, no significant effect in females) [123]. Mouse (*M. musculus*) — 0.1% w/w, 5.8% extension [124]. Also, 100 mg/kg 0f body weight increases lifespan of SHR female mice by 37.8% [125]. In this context, it is important to note that another article showed that the time of metformin administration (in the same concentration and mice strain) is of great importance. when metformin is given from 3 months of the mouse's life there is an increase in 14.1%, from 9 months, 6.2%, and from the 15th month there is no significant change [126].	Fly (*D. melanogaster*) — Decreases the accumulation of ubiquitinated protein aggregates in flight muscles [122]. Killifish (*N. Guentheri*) — Enhances locomotory activity. Memory, and learning. Moreover, it decreases brain inflammatory response, senescence, and degeneration [123]. Mouse (*M. musculus*) — Improved fitness and significantly reduced lens opacity of ∼2Y-old-mice [124]. However, metformin does not decrease spontaneous tumor incidence in female SHR mice [125].	Nematode (*C. elegans*) — PRDX-2 [119]. Fly (*D. melanogaster*) — The decreased accumulation of ubiquitinated protein aggregates in flight muscles is AMP-activated protein kinase (AMPK)-dependent [122].
NAD+ (dinucleotide, natural)	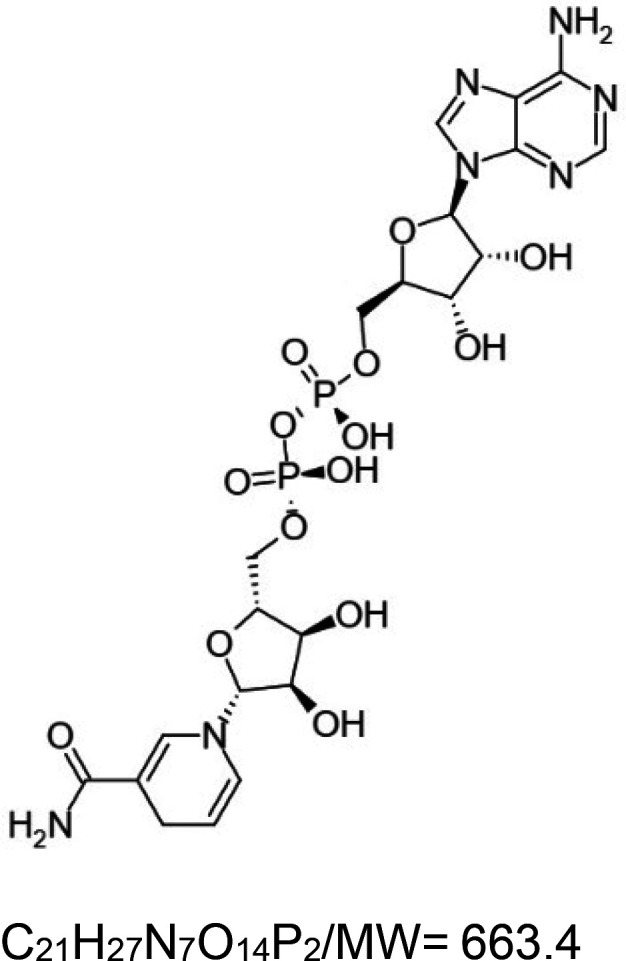	Yeast (*S. cerevisiae*) — treated with 10 µM NAD+ precursor, i.e. nicotinamide riboside (NR) — 10.6 generation extension [127]. Nematode (*C. elegans*) — N2 (wild type) worms and *atm-1(gk186)* mutants treated with 500 µM NR– 20% [128] and 13% extensions [129], respectively. Mouse (*M. musculus*) — treated with either 0.5 and 1.0 g Nicotinamide (NAM)/kg of diet, NS (males) [130]. Also, all of the vehicle-treated *Atm*^−/−^ mice passed by the age of 5 months. In contrast, 80% of the NR-fed *Atm*^−/−^ mice (12 mM) survived over 10 months [129].	Yeast (*S. cerevisiae*) — treated with 10 µM NAD+ precursor, i.e. nicotinamide riboside (NR) — 10.6 generation extension [127]. Nematode (*C. elegans*) — N2 (wild type) worms and *atm-1(gk186)* mutants treated with 500 µM NR — 20% [128] and 13% extensions [129], respectively. Mouse (*M. musculus*) — NR or NMN (nicotinamide mononucleotide) significantly improved locomotory activity and memory of *Atm*^−/−^ mice [129].	Nematode (*C. elegans*) — Life extension requires *sir-2.1* [128]. Moreover, *daf-16* and the mitophagy genes *pink-1*, *pdr-1*, and *dct-1* are required for NR optimum activity [129].
Sodium butyrate (histone deacetylase inhibitor)	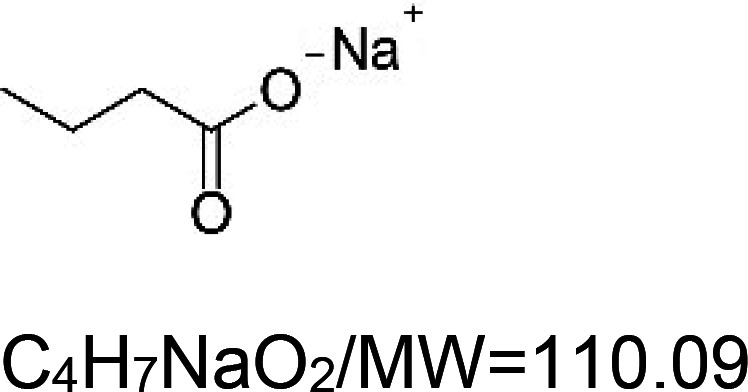	Nematode (*C. elegans*) — 5 mM, 21% extension [131]. Fly (*D. melanogaster*) — 8.5% (20 mM/males) and 6.3% (10 mM/females) [132]. Mouse (*M. musculus*) — High dose of sodium butyrate (1.5 mg per kg) extends the lifespan of Atro-118Q mice (a model for dentatorubral-pallidoluysian atrophy) by 13.7% [133].	Nematode (*C. elegans*) — Sodium butyrate ameliorates the paralysis in CL2006 worms (an Alzheimer's disease model in *C. elegans*) induced by amyloid beta expression in the body wall muscles [131]). Mouse (*M. musculus*) — Low and high doses of sodium butyrate (0.5 and 1.5 mg per kg) significantly improve the locomotory activity of Atro-118Q mice [133].	Nematode (*C. elegans*) — CREB-binding protein 1 (CBP-1) is essential for lifespan extension [131].
Sodium L-lactate	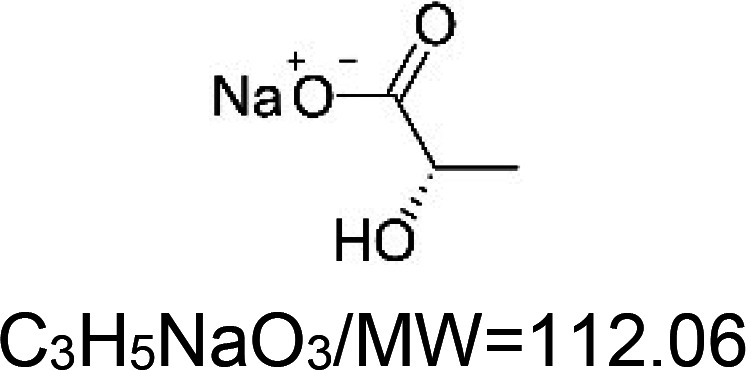	Nematode (*C. elegans*) — 10 mM, 37.5% extension [134].	Mouse (*M. musculus*) — Lactate (117 and 180 mg/kg) improves synaptic plasticity and memory in male mice [135].	Nematode (*C. elegans*) — PMK-1 dependent [134]. Mouse (*M. musculus*) –memory enhancement appears to be mediated by SIRT1 and BDNF [135]
Sodium pyruvate	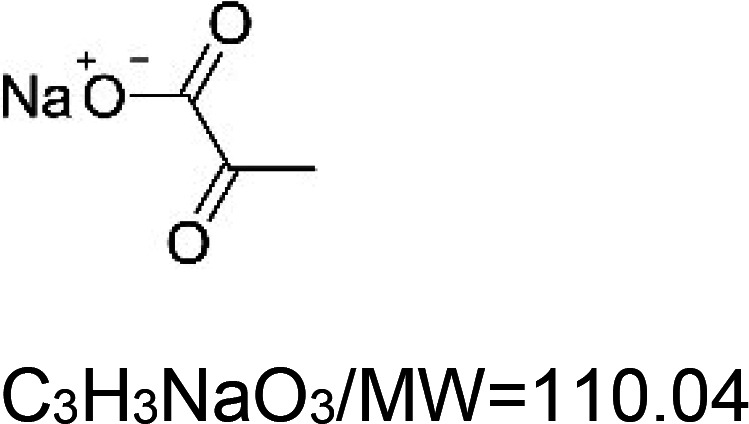	Nematode (*C. elegans*) — 10 mM, 37.5% extension [134].		Nematode (*C. elegans*) — PMK-1 dependent [134].
Uric acid (natural organic acid)	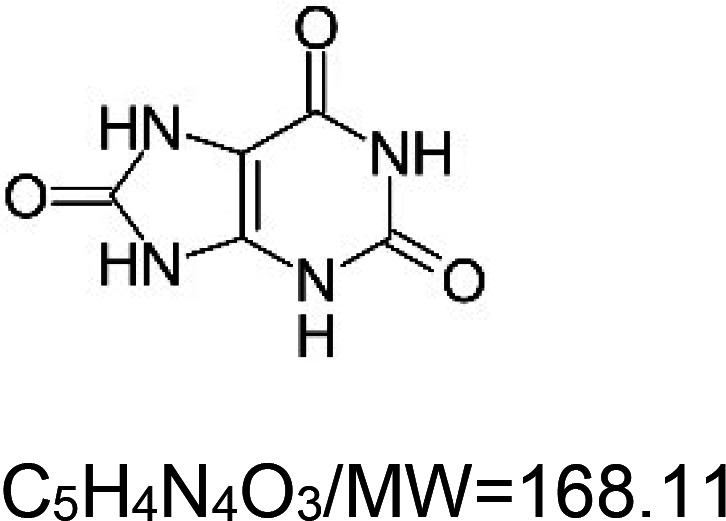	Nematode (*C. elegans*) — 2 mM, ∼14.7% extension [136].	Nematode (*C. elegans*) — 2 mM of uric acid decreases the accumulation of polyglutamine aggregates and increases pharyngeal pumping and movement [136].	Nematode (*C. elegans*) — DAF-16, HSF-1, and SKN-1 are essential for the lifespan extension induced by uric acid [136]. Moreover, mitochondria signals mediated by CLK-1, ISP-1, and MEV-1 are essential. Finally, GLP-1, the *C. elegans* notch homolog, is required for lifespan activity of uric acid [136].
**NEURONAL MODULATORS**				
Cannabidiol (natural phenol)	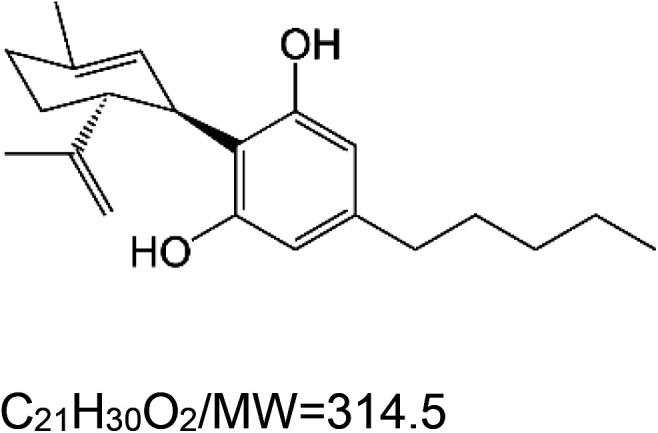	Nematode (*C. elegans*) — 10, 40, and 100 µM extend lifespan by 14.8%, 18.3%, and 12.2%, respectively [137]. Moreover, 1 µM cannabidiol extends lifespan by 23% [138]. Also, 5 µM cannabidiol extends the lifespan by 25.6% and 40% in CL2355 worms (expressing human amyloid beta in neurons) and control CL2122 animals, respectively [139]. Fly (*D. melanogaster*) — 0.1 and 3 µM extend lifespan by 9.94%, and 9.1%, respectively [140].	Nematode (*C. elegans*) — 40 µM enhances mobility in elderly worms [137]. Moreover, 1 µM cannabidiol increases pharyngeal pumping and thrashing [138]. In addition, 5 µM cannabidiol attenuated the deterioration of dopaminergic neurons in CL2355 worms [139]. Also, it enhances pharyngeal pumping activity and chemotaxis in these worms [139]. Fly (*D. melanogaster*) — 0.1 and 3 µM cannabidiol improved the geotaxis response of flies subjected to mild traumatic brain injury (mTBI) [140].	Nematode (*C. elegans*) — The autophagy genes *bec-1*, *vps-34*, and *sqst-1* are essential for lifespan extension. Also, *sir-2.1* and *aak-2* are essential [138].
Lithium	Li/MW = 7.0	Nematode (*C. elegans*) — In wild-type worms, 10 mM Li extends lifespan by 36% (median) [141]. Moreover, the same Li concentration extends the average lifespan of *glp-1(q224)* mutants by 10% [142]. Finally, 10 µM Li extends lifespan by ∼3.5% [143].	Nematode (*C. elegans*) — 10 mM Li attenuates the age-associated locomotory decline of *glp-1(q224)* worms [142]. Fly (*D. melanogaster*) — 1–25 mM concentration range, 16% extension (median, females) [144].	Nematode (*C. elegans*) — *gsk-3*β is required for lifespan extension in wild-type worms [141]. Fly (*D. melanogaster*) — Lifespan extension involves the suppression of the glycogen synthase kinase-3 (GSK-3) and the induction of NRF-2 [144].
Rilmenidine (antihypertensive agent)	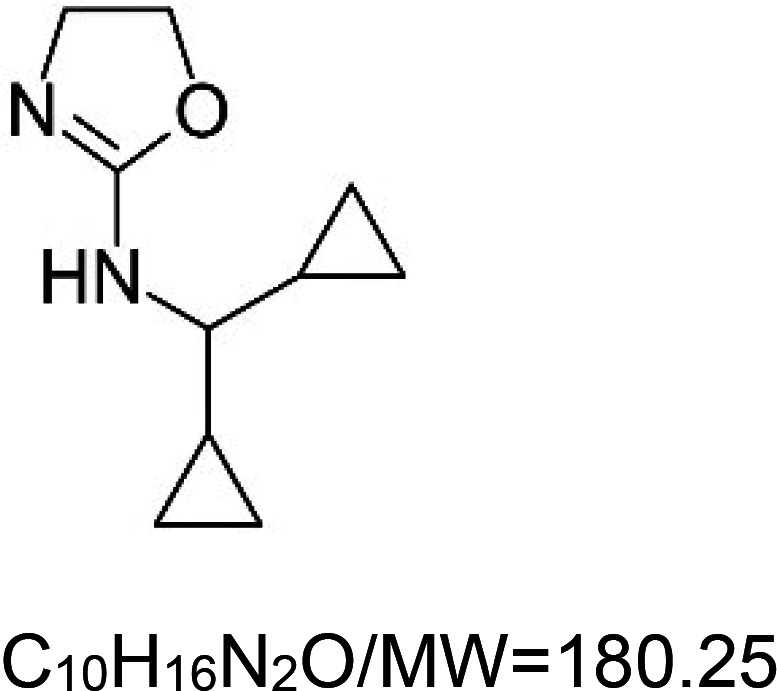	Nematode (*C. elegans*) — 200 µM, ∼19% extension [145].	Nematode (*C. elegans*) — Rilmenidine significantly decreases the accumulation of polyglutamine aggregates (in animals expressing polyQ40). In addition, it preserves the locomotory activity of elderly worms [145]. Mouse (*M. musculus*) — 1 mg/kg rilmenidine (four times a week) worsen motor dysfunction and neuron degeneration in TDP-43WTxQ331K mice [146].	Nematode (*C. elegans*) — DAF-16 and SKN-1 are required for the rilmenidine lifespan extension. Moreover, the autophagy genes *bec-1* and *lgg-1* are required. Finally, NISH-1 (the *C. elegans* ortholog of human nischarin) is also essential for the lifespan activity of rilmenidine [145].
**AUTOPHAGY MODULATORS**				
Rapamycin (an immunosuppressant drug)	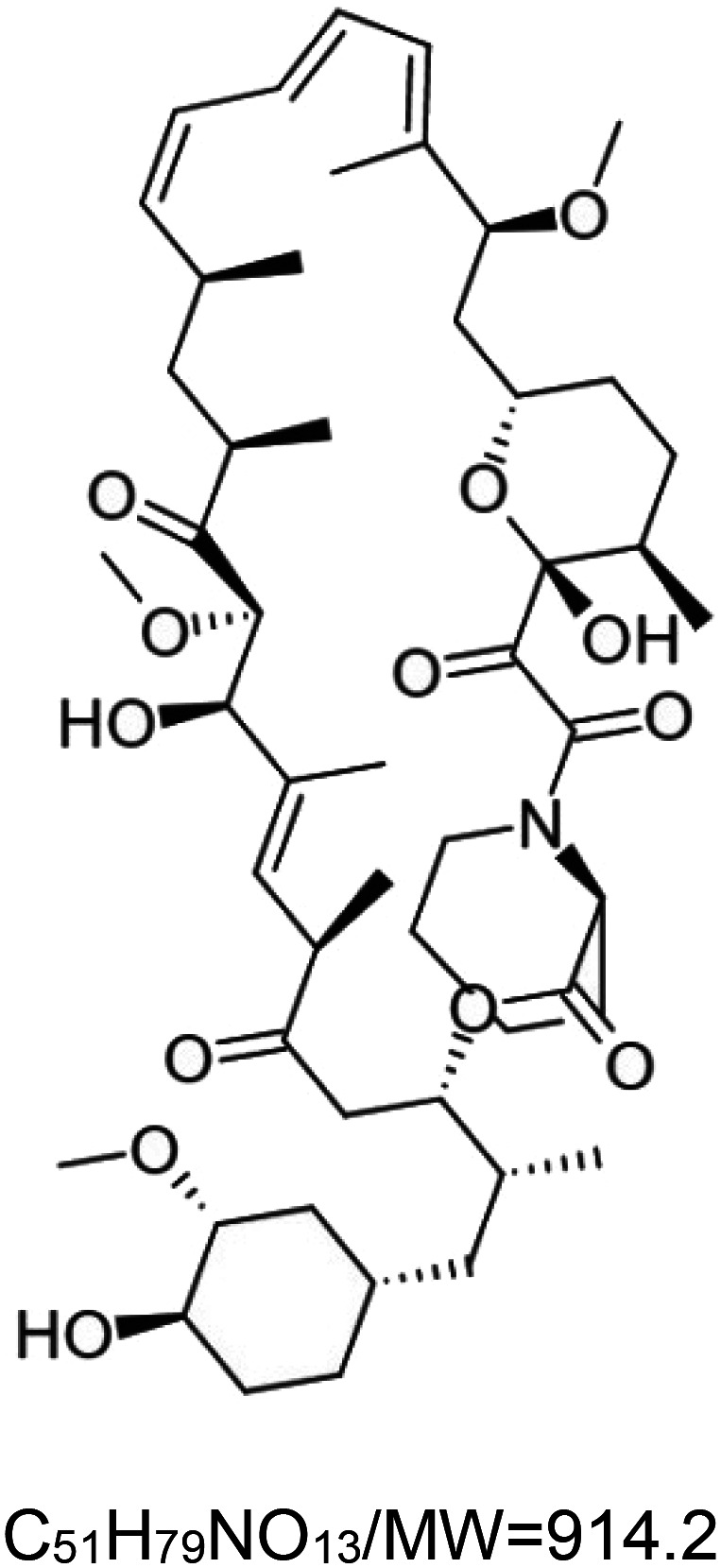	Yeast (*S. cerevisiae*) — 1 ng/ml, 54% extension (with respect to the integrals under the life span curves of the vehicle control) [147]. Nematode (*C. elegans*) — 100 µM, 19% extension [148]. Fly (*D. melanogaster*) — 50, 200, 400 µM result in 3%, 10%, and 7% increase in females, respectively. And, 200 µM increases male lifespan by 6% [149]. Mouse (*M. musculus*) — *Males* — Injection (8 mg/kg/day, 53% extension); Feeding (126 ppm, 21%). *Females* — Injection (8 mg/kg/day, -3% extension); Feeding (126 ppm, 37%). *Notably*, rapamycin was given from the age of 23–24 months [150]. Mouse (*M. musculus*) — 14 mg/kg of food, 10% and 18% extension in median lifespan in *males* and *females*, respectively [91].	Mouse (*M. musculus*) — enhanced forelimb grip strength and Rotarod performance [150].	Yeast (*S. cerevisiae*) — the autophagy genes *atg1* and *atg7* are required for life span extension by rapamycin [151]. Nematode (*C. elegans*) — SKN-1 is required for lifespan extension [148].
Spermidine (polyamine)	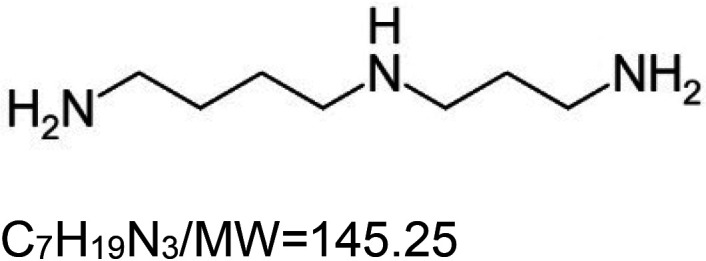	Yeast (*S. cerevisiae*) — 4 mM, up to four times In comparison with vehicle control [152]. Nematode (*C. elegans*) — 200 µM, 15% extension [152]. Fly (*D. melanogaster*) — 1 mM, 30% extension [152]. Mouse (*M. musculus*) — 0.3 mM (drinking water), 10.5% extension when given from the age of 4 months [153]. Rat (*Rattus norvegicus*) — 25 mg/kg/day of spermidine (given to 18 months rats, middle age) does not affect lifespan (nor median or maximal) [154].	Mouse (*M. musculus*) — 3 mM (drinking water) improved cardiomyocyte activity in elderly animals, reflected by mitochondrial and myofibrillar volumes [153]. Rat (*Rattus norvegicus*) — 25 mg/kg/day of spermidine (given to 18 months rats, middle age) decreases anxiety and enhances exploratory behavior. Moreover, it decreases neuroinflammation [154].	Mouse (*M. musculus*) — cardioprotection is dependent on the *Atg5* autophagy gene [153].
1,8-Diaminooctane (VL-004)	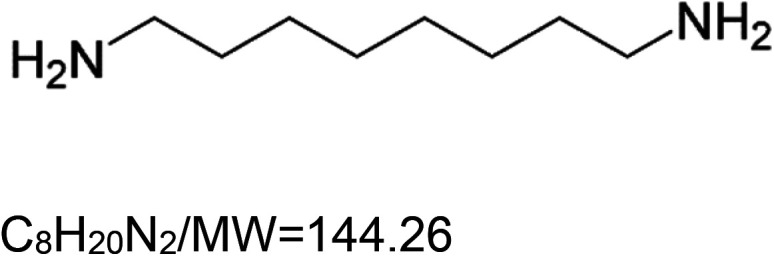	Nematode (*C. elegans*) — 0.25 mM and 4 mM result in 20.6% and 21.8% extensions, respectively [155].	Nematode (*C. elegans*) –4 mM VL-004 significantly increases muscle mass, locomotory, and mitochondrial activity in elderly worms (11 days post-L1). In addition, the same concentration ameliorates paralysis induced by polyglutamine. Finally, VL-004 significantly protects against motor activity deterioration in a worm model of ALS disease [155].	Nematode (*C. elegans*) — The activity of VL-004 requires the function of the mitophagy proteins PINK-1 and DCT-1, as well as the autophagy protein SQST-1/p62. Moreover, it requires the activities of the DAF-16, HIF-1, HLH-30, PHA-4, and SKN-1 [155].
*O*,*O*′-(octane-1,8-diyl)bis(hydroxylamin) VL-850^5^	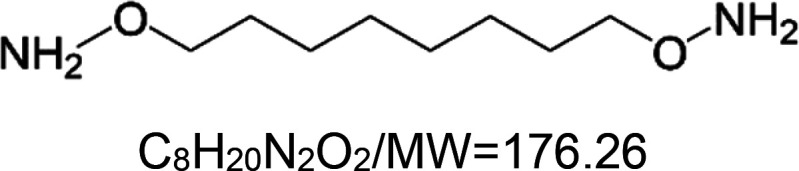	Nematode (*C. elegans*) — 15.62 µM and 62.5 µM result in 20.6% and 36.1% extensions, respectively [155].		
**ANTIBIOTICS**				
Doxycycline hydrochloride	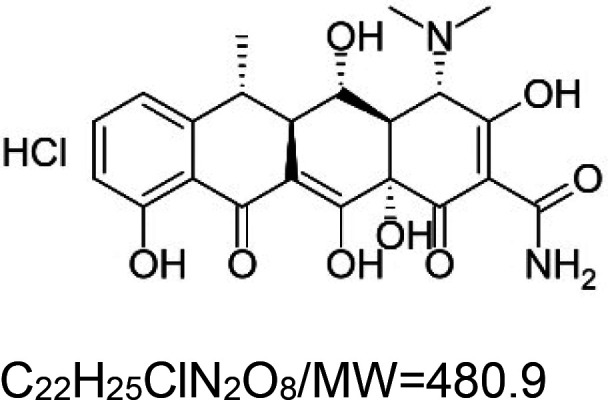	Nematode (*C. elegans*) — 33 µM, 18% extension [156]. Mouse (*M. musculus*) — 5000 and 8000 ppm of doxycycline increases the median lifespan of *Ndufs4−/−* mice by 74% and 84%, respectively [157].	Mouse (*M. musculus*) — 5000 and 8000 ppm of doxycycline improve rotarods’ performance of day 40 and day 50 *Ndufs4−/−* mice [157].	
**ANTIFUNGAL AGENT**				
Ciclopirox olamine	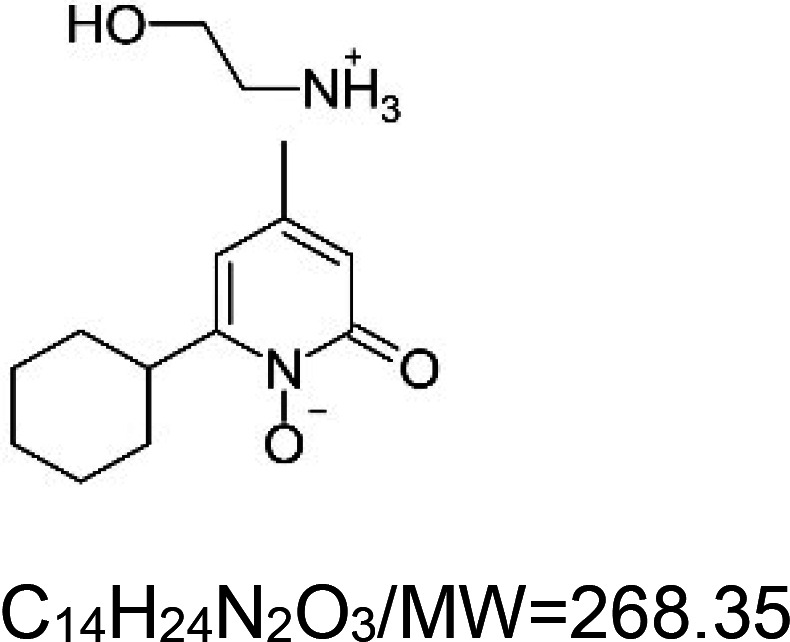	Nematode (*C. elegans*) — 0.01% (∼373 µM), 41% extension [51].		
**ANTIPARASITIC AGENT**				
Ivermectin	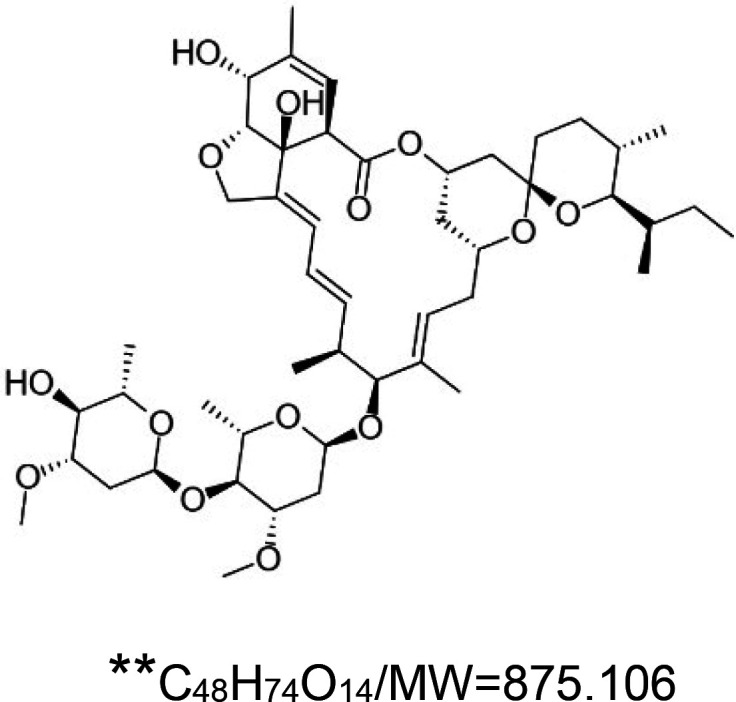 ^3^	Rotifer (*B. manjavacas*) — 1 µM, 21% extension [158]. Nematode (*C. elegans*) — 0.1 pg/ml and 1 pg/ml, 21% and 22% extension, respectively [159].		Nematode (*C. elegans*) — DAF-16 dependent [159].
**REACTIVE OXYGEN SPECIES GENERATOR**				
Paraquat	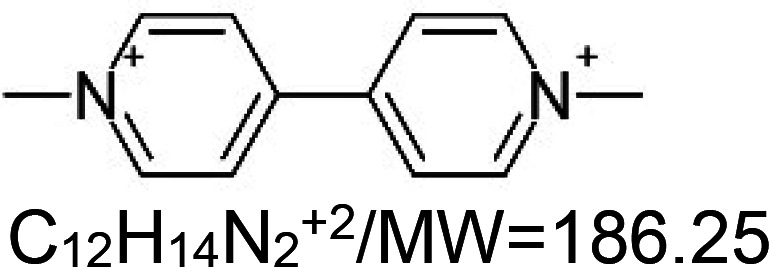	Nematode (*C. elegans*) — 0.1 mM, 58% extension [160].		
Sodium arsenite	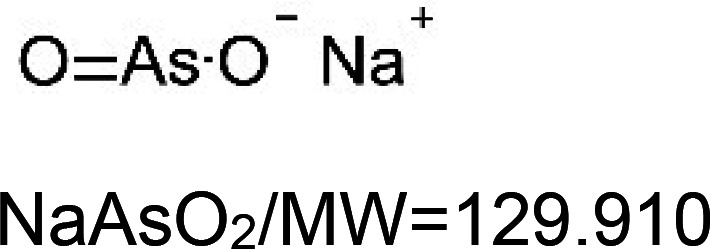	Nematode (*C. elegans*) — 0.1 µM, 9.5% extension [161].		Nematode (*C. elegans*) — DAF-16 and SKN-1 dependent [161].

1 In cases where the change in the average lifespan was not reported, we indicate the relevant reported measurement, e.g. the change in median lifespan;

2 FW1256 and GYY4137 are not natural substances but synthetic hydrogen sulfide donors [162];

3 Ivermectin has several structures/MW [163];

4 Lifespan effect in *C. elegans* depends on *E. coli* folate metabolism [120];

5 VL-850 is a newly developed compound that has yet to be extensively characterized. However, it exhibits a chemical structure highly similar to VL-004. In addition, like VL-004, VL-850 also protects against oxidative stress and prolongs the lifespan of *C. elegans* [155]. Based on these similarities, we hypothesize that the two substances operate through a similar mechanism of action.

An important clarification must be made regarding Table 1. The table showcases substances that activate mitophagy and potentially extend lifespan and/or healthspan. It is important to acknowledge that the data for these two aspects may originate from separate studies. Furthermore, in many instances, a proven causal relationship has not been established between a substance's impact on mitophagy and its ability to prolong life or healthspan. In simpler terms, while strong evidence might suggest that a specific substance induces mitophagy and extends lifespan or healthspan, the direct cause-and-effect link between these functions remains unverified. Given this context, the data presented in Table 1 offers an opportunity for further investigation into the causal relationship between substance activity in mitophagy and life/healthspan extension.

**Figure 2.**
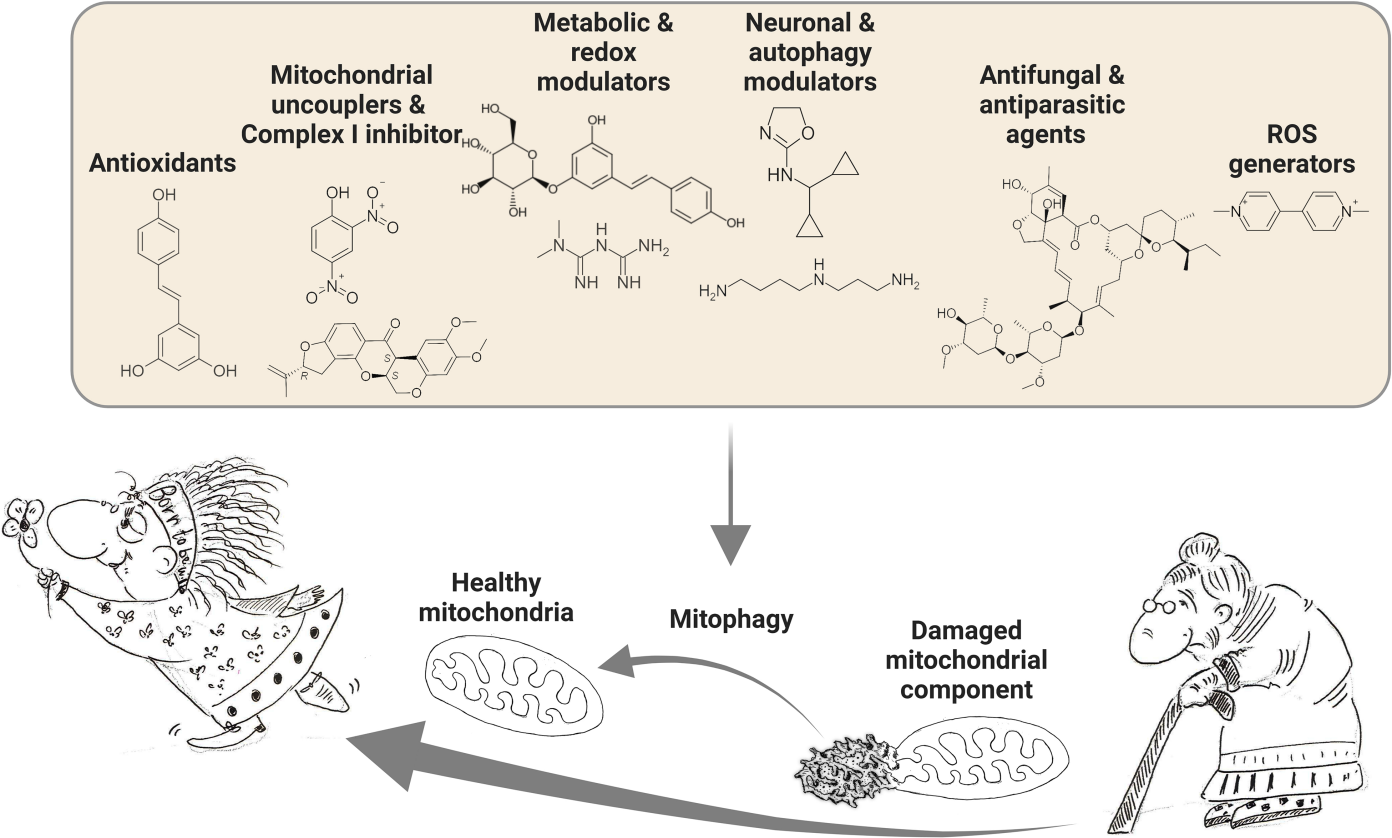
Healthy mitophagy activation by natural and synthetic substances. A variety of natural and synthetic substances induces mitophagy. Selectively removing damaged mitochondria ensures cellular health and presents an emerging therapeutic strategy to promote healthy aging.

We classify each substance based on its biological activity. This is has been a challenging task, because most substance (or even all) have more than one biological activity. However, for the sake of clarity and readability, we have assigned each substance to a specific category. Additionally, we acknowledge that some readers may be interested in knowing whether a substance is of natural or synthetic origin. Hence, we have explicitly indicated the natural origin for all substances. We classified the mitophagy activating substances into the following categories: antioxidants, mitochondrial uncouplers, complex I inhibitors, redox, metabolic, neuronal, autophagy modulators, antibiotic and ant-fungal agents, and reactive oxygen species (ROS) generators (Table 1 and Figure 2).

It is worthwhile noting that many of the natural substances that trigger the mitophagy pathway are *polyphenols*. Polyphenols are a diverse group of naturally occurring compounds found abundantly in plants and are considered to be the most widely distributed phytochemicals among all plant-based sources. Collectively, they have gained significant attention due to their health-promoting properties, including lifespan extension [164,165]. The longevity-promoting effects of polyphenols depend on the structural characteristics of their carbocyclic rings and the number of hydroxyl groups present on the ring [68]. Studies in *C. elegans* suggest that polyphenols act through specific longevity pathway proteins, i.e. the DAF-16/FoxO3 and SKN-1/NRF2 TFs and the SIR-2.1/SIRT1 sirtuin [166]. These proteins appear to mediate the life-extension activity of the polyphenol mitophagy activators baicalein [53], catechinic acid [167], curcumin [58], myricetin [76], naringin [78], resveratrol [87], and urolithin A [95].

Intriguingly, DAF-16 and SKN-1 regulate the activity of the mitophagy receptor DCT-1 (the *C. elegans* homolog of the mammalian BNIP3 and BNIP3L/NIX) [168]. In this regard, it is important to indicate that mitophagy activating via DCT-1 protects from oxidative injury [155]. Many dietary polyphenols are potent antioxidants [165]. However, whether their bioavailability through the digestive system and effective transport to cells facilitate such function *in vivo* is still a matter of controversy [169,170]. Thus, a plausible hypothesis is that the antioxidant activity of polyphenols is partly mediated indirectly, such as through the activation of mitophagy [171].

Many of the synthetic substances are drugs, with some being FDA-approved., i.e. aspirin, doxycycline hydrochloride, ivermectin, metformin, and rapamycin. Other substances are potent ROS inducers (paraquat and sodium arsenite), Uncouplers of oxidative phosphorylation (2,4-dinitrophenol and CCCP), Metabolism modulators (dichloroacetate and metformin), and Synthetic diamines (VL-004 and VL-850).

Importantly, as indicated for the natural substances, some compounds could be classified in more than one group. For example, CCCP is a potent mitochondrial uncoupler and (thus) inducer of oxidative stress [172]. However, we chose to assign each material to a single category for simplicity.

Similar to the natural substances, several synthetic ‘substances’ activities are mediated by DAF-16 and SKN-1 TFs; for instance, ivermectin, sodium arsenite, aspirin, rapamycin, rilmenidine, 1,8 diaminooctane (VL-004), and others. Our studies on the synthetic diamine VL-004 show that, at least in *C. elegans*, additional TFs are needed for mitophagy-dependent antioxidant activity. That is, the hypoxia-inducible factor 1 (HIF-1), HLH-30/TFEB, and PHA-4/FOXA. Notably, these TFs are known to regulate/modulate lifespan and healthspan in *C. elegans* [173–176], further supporting the causative function of mitophagy in lifespan regulation. A major challenge is understanding how these TFs activities are coordinated to extend life- and healthspan.

### Could activation of the mitophagy process using a mitophagy activating compound (MAC) cocktail increase the medical benefit

Previous studies suggest that drug combinations may elicit a therapeutic effect greater than the sum of the individual drugs [177]. Indeed, there are evidence that targeting different components of the same cellular/physiological process may be advantages [178]. An example of such synergistic activity between two drugs acting on the same target is the anticancer combination of cisplatin and trabectedin. Trabectedin interacts with DNA and DNA repair systems differently than cisplatin does. Specifically, trabectedin inhibits DNA replication through a different mechanism of action, which decreases the antagonistic activity against cisplatin, resulting in a synergistic effect [178]. To the best of our knowledge, no systematic research has been conducted to examine the combined impact of mitophagy-activating substances on lifespan and health in any model system.

Nevertheless, despite the medical potential inherent in the MAC cocktail strategy, we would like to point out a possible limitation. A recent article we published showed that the synthetic diamine VL-004 extends the health and lifespan of *C. elegans* [155]. Moreover, we demonstrated that VL-004 protects *C. elegans* and human-derived cell lines from oxidative injury. This protective activity depends on the TF DAF-16, which DAF-2 negatively regulates; the *C. elegans* sole ortholog of the mammalian insulin and IGF-1 receptors (IIR) [179]. DAF-2 inhibits DAF-16 from entering the nucleus. As a result, when the activity of DAF-2 is suppressed, DAF-16 can move into the nucleus, where it activates genes crucial for stress resistance and increased lifespan. Among these genes are antioxidant genes (i.e. catalases 1 and 2 and superoxide dismutase 3 [180]), and the TF SKN-1 activates multiple antioxidant genes [181]. Therefore, it is unsurprising that worms bearing loss-of-function mutations impairing DAF-2 activity (*daf-2* mutants) are more resistant to oxidative stress [182].

**Figure 3.**
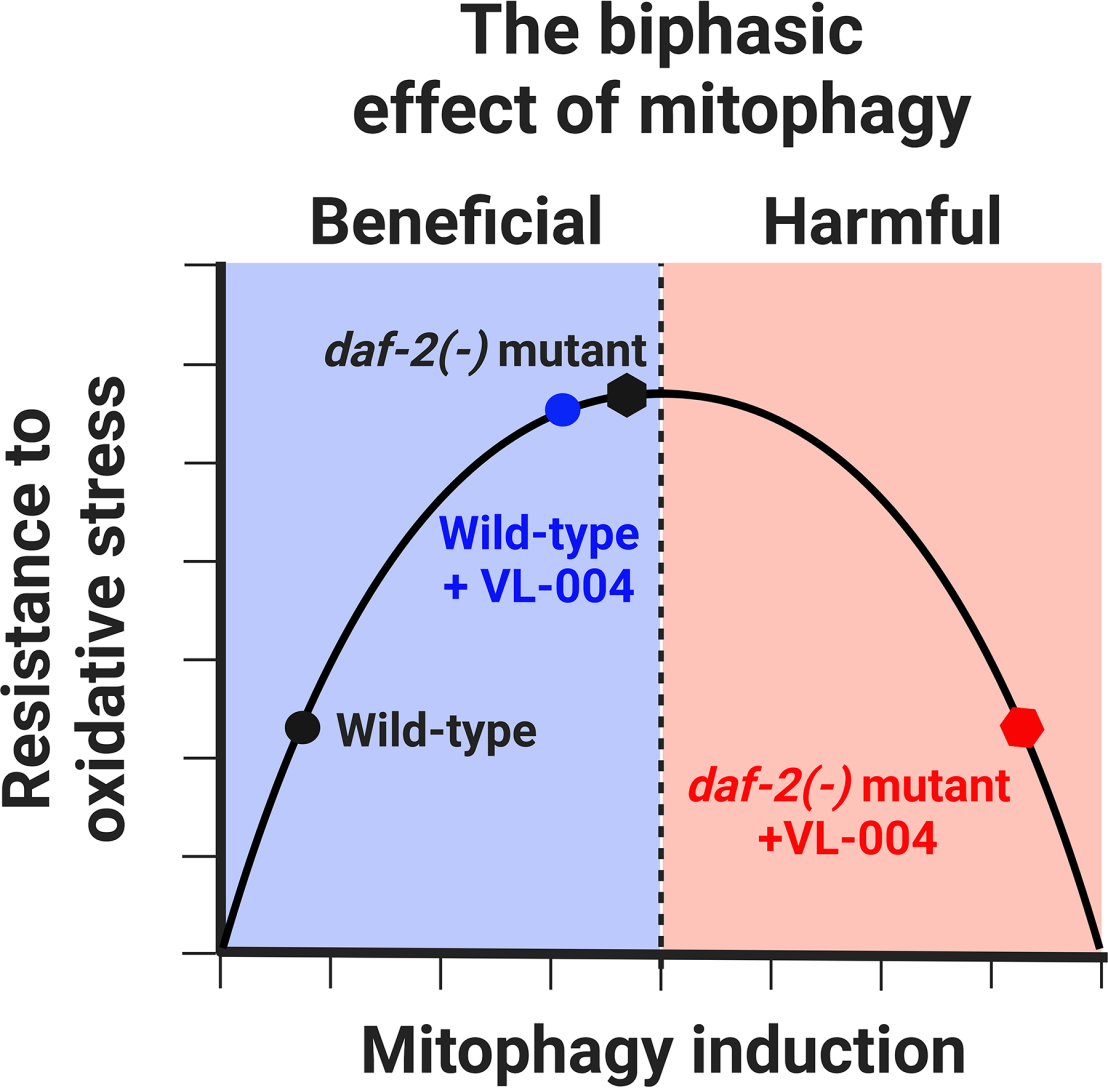
The biphasic effect of mitophagy. The basal level of mitophagy in wild-type worms is significantly lower than in *daf-2* mutants. Similarly, their resistance to oxidative stress is also lower, as represented by the dark circle and hexagon, respectively. Treatment with VL-004 increases the level of mitophagy in wild-type worms, moving it towards the optimal range of mitophagy, thereby increasing ‘worms’ survival in oxidative stress. This brings their survival rate similar to that of *daf-2* mutants (represented by the blue circle and dark hexagon). In contrast, treating *daf-2* worms with VL-004 leads to excessive mitophagy, moving it toward the harmful range. As a result, the survival rate of the *daf-2* animals decreases (represented by the red hexagon) to the level of wild-type worms before the VL-004 treatment.

Intriguingly, VL-004 does not increase the resistance of *daf-2* mutants to oxidative stress [155]. On the contrary, it decreases the resistance to wild-type worms level [155]. A hypothesis that may explain the above observation is that an optimal level of mitophagy maintains an efficient balance of mitochondrial activity and ROS level. Beyond this level, the activation of mitophagy is harmful, perhaps because it eliminates intact mitochondria and/or interferes with healthy ROS signals (Figure 3). Indeed, direct mitophagy measurements show that the basal level of mitophagy in *daf-2* mutants is high, similar to that of wild-type worms treated with VL-004 [155]. As predicted by the hypothesis above, VL-004 increases mitophagy in *daf-2* mutants, driving it into the unhealthy mitophagy zone. In line with this observation, there are evidence that excessive mitophagy can be harmful. For instance, increased mitophagy can lead to the development of heart failure condition due to cardiac myocytes loss [183]. Moreover, it has been suggested that exaggerated mitophagy has a causative role in the pathophysiology of Huntington's diseases [184,185], as well as in stroke and multiple sclerosis [186]. In conclusion, mitophagy is like walking the tightrope, i.e. it requires precise balance. Hence, a treatment plan for the MAC cocktail should be designed not to surpass the healthy mitophagy threshold.

### Do mitophagy-activating compounds demonstrate effectiveness in treating age-associated medical conditions?

To address this question, we explored the https://clinicaltrials.gov/ database, focusing on studies that met the following criteria. Firstly, we solely considered trials involving individuals aged 60 years and above, both male and female. The choice of this population section is driven by the article's topic, namely, improving life expectancy and health. Secondly, our inclusion criteria encompassed both ongoing and completed trials. In situations where multiple studies (>3) were available, priority was given to completed and published trials to ensure comprehensive data availability. Thirdly, our analysis was limited to compounds used in their original form within the study. This criterion effectively eliminated any confounding effects caused by extracts or compound combinations, thus enhancing the clarity of the results and simplifying interpretation. Fourthly, we detailed potential limitations associated with specific treatments, as indicated in the https://clinicaltrials.gov/ database. These potential limitations include factors such as the number of patients participating in the trial, the duration of the treatment, among others. Additionally, we highlighted any observed side effects. Lastly, our research focused on diseases predominantly affecting the elderly and exhibiting a strong correlation with mitophagy decline. By adhering to these criteria, we aimed to offer reliable insights into the existing dataset concerning mitophagy-activating substances within clinical contexts. The results of these studies are outlined in Table 2.

**Table 2:** Effects of mitophagy activators in clinical trials targeting elderly-related conditions

Mitophagy enhancer	Title of the study (NCT number)	Conditions	Intervention and dosage	Tested parameters	Results	Side effects and limitations of the study (if specifically indicated in the clinical report)
Curcumin	Curcumin and function in older adults (NCT03085680)	Physical functions and cognitive functions in old age	Dietary Supplement: Curcumin: 1000 mg/day for 3 months.	Physical Function — Walking and hand grip, cognitive Function — Attention & Memory, pain, inflammation — Interleukin-6	Curcumin improves physical, cognitive, and immune functions.	
	Effect of curcumin on microvascular response and tissue oxygenation (NCT04119752)	Cardiovascular risk factor	Dietary Supplement: Curcumin powder: 10 g, and studies were conducted after 2 h of administration.	Changes in microvascular reactivity, tissue oxygen saturation, and nitric oxide metabolites (plasma nitrate and nitrite)	Curcumin supplementation improves the older population's pre-frontal cortex oxygenation and blood volume [187].	
Resveratrol	Impact of resveratrol on brain function and structure (NCT02621554)	Healthy	Dietary Supplement: Resveratrol: 200 mg/day for 26 weeks.	Change from baseline verbal learning task scores at 6 and 12 months, mini-mental state examination, functional changes on brain MRI images and plasma biomarkers	No significant difference in the treatment versus placebo [188]. Increase in serum cholesterol and overall health decline in both treatment and placebo.	Further studies with extended duration of the drug treatment and larger sample size would be required for more conclusive results.
	Cardiovascular health of older adults and resveratrol (CORE) (NCT02909699)	Aging	Dietary Supplement: Resveratrol: 1000 mg of resveratrol per day (one pill three times daily). Or 1500 mg of resveratrol per day (one pill three times per day).	Speckle tracking analyses, flow-mediated dilation, autophagy, differential endothelin-1 protein expression, endothelial function endothelial nitric oxide synthase (eNOS).	No results available	
Urolithin A	Bioenergetics and muscle function improvement with AMAZ-02 in elderly skeletal muscle (ENERGIZE Trial) (NCT03283462)	Mitochondrial function, bioenergetics, muscle functions in Aging	Dietary Supplement: Mitopure (Urolithin A): 1000 mg/day, and the studies were conducted at the baseline (start of the study, 2 months and 4 months).	Change in 6 min walking distance (6MWD), percent change from baseline in ATP max (maximal ATP synthesis rate) in hand skeletal muscle, percent change from baseline in contraction number during a hand muscle fatigue test, percent change from baseline in ATP max (maximum ATP synthesis rate) in leg skeletal muscle, percent change from baseline in contraction number during a leg muscle fatigue test, changes in Short Physical Performance Battery (SPPB) scores at the end of the study intervention compared with baseline, change in exercise tolerance compared with baseline (via cycle ergometry), change in hand grip strength, change in leg muscle strength (1-RM and 10-RM), change in muscle size (cross-sectional area of the muscles), changes in mitochondrial function on muscle biopsy samples, effect of AMAZ-02 on mitochondrial gene and protein expression in muscle tissue, the effect of AMAZ-02 on plasma acylcarnitines, the effect of AMAZ-02 on quality-of-life questionnaire (SF36), change from baseline in plasma lipid profile and change from baseline in plasma for circulating biomarkers (myostatin, follistatin)	Urolithin A is overall safe for consumption. 6MWD and ATP production in hand skeletal muscle did not significantly differ between the placebo and the treatment. However, long-term treatment benefitted muscle endurance and plasma biomarkers [189].	In this study, overall, there was an increase in both placebo and drug for changes in 6MWD, which may be because the participation in this study motivated the participant to increase their daily activity, and daily participant physical activity was not monitored. Secondly, the study was carried out in a small sample size and only in the white population from Seattle, Washington area and thus not applicable to all elderly population [189].
Nicotamide riboside (NR)	The effects of nicotinamide adenine dinucleotide (NAD) on brain function and cognition (NCT02942888)	Mild cognitive impairment	Dietary Supplement: Nicotinamide riboside: 250 mg (week 1), 500 mg (week 2), 750 mg (week 3), 1g (weeks 4–10).	Changes in Montreal Cognitive Assessment (MoCA), cerebral blood flow, plasma NAD, Short Physical Performance Battery (SPPB), Instrumental Activities of Daily Living (IADLs), endothelial function, Geriatric Depression Scale (GDS), Geriatric Anxiety Scale (GAS), Clock Drawing Task Protocol (CLOX), Executive Interview (EXIT), Test of Auditory Processing Skills (TAPS) and grip strength from baseline at 10 weeks	No results available	
	Inflammation in COPD and the effect of nicotinamide riboside (NCT04990869)	COPD	Dietary Supplement: Nicotinamide Riboside: 2000 mg/day	Changes in Interleukin-8, NAD+ levels, interleukin-6, interleukin-10, Tumor necrosis factor alpha, C-reactive protein and matrix metalloproteinase-9	No results available	
	Slow age: interventions to slow aging in humans (NCT05593939)	Aging	Dietary Supplement: Nicotinamide riboside: 2000 mg/day for 6 weeks	Changes in Interleukin-6, CRP, TNF-α, NAD levels in plasma, change in fat and lean mass, grip strength, gait speed, predicted age by studying DNA methylation, Blood age, transcriptomics, voice, and photographs of the patient.	No results available	
	NR supplementation and exercise (NCT04907110)	Overweight, obesity and type 2 diabetes in aging	Niagen: 1000 mg/day. Studies were conducted three days prior to NR supplementation and after 40 days of treatment.	Changes in *ex-vivo* muscle mitochondrial function, maximal aerobic capacity, physical performance, skeletal muscle NAD concentrations (ex-vivo and *in vivo*), intrahepatic lipid content, upper leg muscle mass, body composition, quality of life, blood metabolites, submaximal exercise energy expenditure, sleeping metabolic rate and exercise efficiency	No results available	
Cannabinoid	The effect of cannabis on dementia-related agitation and aggression (NCT03328676)	Agitation related to dementia	Avidekel oil drops given under the tongue: that contains 30% cannabidiol and 1% tetrahydrocannabinol: 295 mg and 12.5 mg per ml three times a day for 16 weeks)	Changes in score of the Cohen-Mansfield Agitation Inventory (CMAI) and in the score of the Neuropsychiatric Inventory-Nursing Home Version (NPI-NH)	The use of ‘Avidekel’ oil resulted in a notable decrease in agitation compared with the placebo in patients experiencing behavioral disturbances associated with dementia [190].	Side effects may include drowsiness. The study had limitations due to a small and heterogenous participant group from a single medical center. It didn't compare different dementia sub-types, affecting the safety profile assessment of the product containing THC. Functional impairment measures and pharmacokinetic indices were missing. A much extensive study with a larger sample size is required.
Astaxanthin	Effect of astaxanthin on the patients with Alzheimer's disease (NCT05015374)	To evaluate the possible benefit of astaxanthin on Alzheimer's disease	Dietary Supplement: Astaxanthin (350 mg/day) for 3 years.	Mini-Mental State Examination (MMSE), Cognitive Ability Screening Instrument (CASI), Clinical Dementia Rating (CDR), Neuropsychiatric Inventory (NPI), and incidence of treatment-emergent adverse events (safety and tolerability)	No results available	
Metformin	Metformin to augment strength training effective response in seniors (MASTERS) (NCT02308228)	Aging	Behavioral: Progressive Resistance Training. Drug: Metformin 1700 mg/day for 16 weeks	Percent change in type 2 myofiber cross sectional area (using the muscle biopsies of the vastus lateralis) and percent change in normal density muscle size by computed tomography	Metformin has a detrimental effect on the hypertrophic response to resistance training in healthy older individuals. The study showed that participants who received the placebo experienced significantly greater gains in lean body mass (*P* = 0.003) and thigh muscle mass (*P* < 0.001) compared with those who took metformin [191]. Patients who had higher levels of muscle fat (lipid) at the beginning of the study showed reduced gains in muscle mass and strength when they took metformin. Similarly, the individuals who experienced the greatest changes in muscle fat also had limited improvements in strength. This suggests that for people to get the most benefit from strength training exercises, their muscle metabolism needs to adapt and reduce fat content. However, metformin seems to interfere with this adaptation process, leading to less effective results from both aerobic and strength exercises [192]..	Side effects may include nausea, diarrhea, and flatulence. This study focused on non-obese individuals who were free from any metabolic disease. It excluded the sedentary group.
	Metformin and Muscle in Insulin-resistant Older Veterans (NCT01804049)	Prediabetes	Drug: metformin 850 mg/day for 1 month	Changes in total lean mass from baseline, in physical performance - 400 m walk speed and muscle characteristics	Preliminary data suggests older individuals who have diabetes experience a faster decline in muscle mass and gait speed unless they are undergoing treatment with metformin. Moreover, older adults with prediabetes exhibit a more significant reduction in muscle mass and a higher likelihood of experiencing disability.	Side effects may include chest pain, nausea, diarrhea, weight loss, and memory loss.

## When it comes to mitophagy, ‘one size doesn't fit all’

Dysfunctional mitophagy is implicated in the pathophysiology of multiple age-associated maladies, including Alzheimer's and Parkinson's diseases, cardiovascular diseases, and sarcopenia [193,194]. Therefore, enhancing mitophagy is a promising strategy for future disease treatment [195]. However, there are cases in which mitophagy can contribute to disease development. For example, functional mitophagy appears to be necessary for tumor progression. In tumors with oncogenic KRAS mutations, mitophagy increases malignancy [196]. Indeed, Selective inhibition of autophagy/mitophagy by liensinine, a natural alkaloid, increases the cell death activity of doxorubicin through dynamin 1-like (DNM1L)-dependent mitochondrial fission [197]. Similarly, mdivi-1, a DRP1-mediated mitophagy inhibitor, sensitizes hepatic cancer cells to cisplatin and enhances cancer cell death [198].

Apart from cancer, several pathogenic viruses (e.g. CSFV, HBV, HCV, and MeV) activate mitophagy to escape cell death, thereby promoting virus infectivity. For instance, measles virus vaccine strain Edmonston B (MV-Edm) infection activates mitophagy, leading to a decrease in the release of somatic cytochrome C (CYCS). As a consequence, apoptotic cell death is inhibited in non-small cell lung cancer cells [199]. In conclusion, while using small-molecule substances to activate mitophagy presents promising therapeutic opportunities, this approach should be carefully considered due to the multiple roles of mitophagy in cellular health and disease.

## The other side of the same coin: mitochondrial biogenesis

As mentioned, the selective elimination of damaged mitochondrial components is essential for the normal activity of the mitochondrial network. However, this step must be accompanied by the supply of new ones in a process called mitochondrial biogenesis [200]. Indeed, precise synchronization of mitochondrial biogenesis and mitophagy is necessary for the cells to adapt to different metabolic states and stress conditions, and a lack of coordination is closely associated with various pathological conditions (e.g. ‘Parkinson's disease) [200].

Since the process of mitochondrial biogenesis is not the subject of this article, we would like to focus on only one point related to the subject. That is, the activation of mitochondrial biogenesis by the same molecules that activate mitophagy and common signaling pathways/molecules.

### Resveratrol

Resveratrol induces mitochondrial biogenesis in several cell types, including brain cells [201,202]. Moreover, it has been shown to have beneficial activity in several disease models, including models associated with accelerated aging. For instance, administering 20 mg of resveratrol per kg of body weight one day before the monocular derivation procedure prevented the decrease in AMP-activated protein kinase (AMPK) phosphorylation, peroxisome proliferator-activated receptor gamma coactivator 1 alpha (PGC-1α), and nuclear respiratory factors 1 (NRF-1) in the visual cortex of rats [203]. Moreover, resveratrol (60 µM, 24 h pretreatment) enhances mitochondrial biogenesis through the functions of PGC-1α and mitochondrial TF A in a cellular model system (PC-12 cells) for ‘Parkinson's disease [204]. *Notably*, NRF-1 (as well as NRF-2), the estrogen-related receptors (α, β, and γ), and PGC-1α are key TFs that control mitochondrial biogenesis in mammalian cells [200]. Lastly, resveratrol (160 mg/kg per day, for 8 weeks) improves cognition and mitochondria function in a mouse model of accelerated aging (senescence-accelerated mouse prone 8, SAMP8) subjected to a high-fat diet [205]. Interestingly, resveratrol increases the level of the mitochondria respiratory complexes without affecting the levels of PGC-1α and SIRT1, however increasing AMPK-phosphorylation.

### Metformin

Metformin (2 mM) administration attenuates human endothelial cell senescence by promoting mitochondrial biogenesis through the activation of AMPK and by increasing the levels of SIRT3 and PGC-1α [206]. Moreover, metformin enhances the trimethylation of H3K79 (H3K79me3) in the SIRT3 promoter region through an SIRT1-DOT1L signaling axis. This, in turn, increases SIRT3 expression, leading to enhanced mitochondrial biogenesis [206]. Furthermore, metformin (1700 mg/day, for at least one year) increases PGC-1α levels in peripheral blood mononuclear cells (PBMCs) derived from type 2 diabetic patients [207], as well as in liver and skeletal muscle cells [208,209].

### At the molecular level

At the molecular level, the activation of AMPK serves as a junction between the pathways of mitophagy activation and mitochondrial biogenesis. Specifically, by activating PGC-1α, AMPK stimulates mitochondrial biogenesis while also triggering autophagy and mitophagy through the unc-51-like autophagy activating kinases 1 and 2 (ULK1/2) [200]. Notably, AMPK is activated by a variety of external/internal stimuli. For instance, elevated calcium (Ca^2+^) levels can activate the Ca^2+^/calmodulin dependent kinase (CaMK), which further activates Ca^2+^-Calmodulin-dependent protein kinase kinase beta (CaMKKβ), and thus AMPK [200,210,211]; CamK can also activate PGC-1 directly.

Despite the abundance of existing knowledge regarding mitophagy and biogenesis processes, there remains a significant knowledge gap in comprehending their interrelationships, especially concerning their control at the organismal level and drug treatment that combines the activation of these processes. Therefore, it would be fascinating to investigate how the combined activation of mitophagy and biogenesis affects the healthspan and lifespan of various biological models.

## Conclusions

Declining mitophagy contributes to cellular damage and the development of conditions such as Alzheimer's, Parkinson's, and cancer. Therefore, stimulating mitophagy via pharmacological intervention presents a potential means to improve cellular health and combat these diseases.

Various types of small molecules have been identified as mitophagy activators, including antioxidants, mitochondrial uncouplers, complex I inhibitors, redox regulators, metabolic modulators, neuronal modulators, autophagy modulators, antibiotic and antifungal agents, and reactive oxygen species (ROS) generators.

In many cases, the activities of these substances are mediated by TFs that orchestrate the cell's response to stresses, such as DAF-16/FOXO, PHA-4/FOXA, HLH-30/TFEB, and SKN-1/NRF-2. However, in many cases, the specific cellular receptors that bind these substances and how this binding triggers the activation of a particular mitophagy pathway remain unclear. Furthermore, the mechanism by which mitophagy activation extends longevity and healthspan is not fully understood. A mechanistic understanding of these processes is crucial for developing more effective and safer molecules to treat various age-related diseases.

Using a mitophagy-activating substances cocktail (MAC) for therapeutic purposes holds great promise in enhancing medical benefits. The MAC cocktail could potentially improve health and lifespan by targeting different components of the same cellular process. However, it is crucial to consider potential limitations, as excessive mitophagy may lead to harmful effects. Maintaining the optimal level of mitophagy is crucial for balancing mitochondrial activity and ROS levels, and exceeding this threshold might have detrimental consequences. Therefore, the design of the treatment plan for the MAC cocktail should carefully consider the delicate balance of mitophagy to ensure it does not surpass the healthy threshold, maximizing its beneficial effects for therapeutic purposes.

Following the previous paragraph, exercising caution in utilizing small-molecule substances to activate mitophagy is essential, as its effects can be context-dependent. Mitophagy's involvement in tumor progression and viral infection demonstrates the dual nature of its impact on cellular processes, where both beneficial and harmful outcomes are observed. Therefore, any therapeutic approach to manipulate mitophagy should be carefully tailored and thoroughly researched to ensure the best possible outcomes in different disease contexts.

Finally, the mitochondrial biogenesis process balances mitophagy to ensure proper mitochondrial network homeostasis. Interestingly, mitochondrial biogenesis may be activated by the same molecules that induce mitophagy, such as resveratrol and metformin. Despite progress in understanding these processes, much remains to be uncovered, particularly in comprehending their interrelationships at the organismal level and exploring combined drug treatments targeting mitophagy and biogenesis. Future research in this area could shed light on how such combined activation may impact healthspan and lifespan in different biological models, potentially paving the way for novel therapeutic strategies in the future.

## Perspectives

- The process of mitophagy is necessary for the activity of the mitochondrial network and deteriorates with age. Thus, one of the leading strategies for treating various aging diseases involving the accumulation of damaged mitochondria, such as Alzheimer's and Parkinson's, is to use small molecules to control and activate the mitophagy process.
- Several natural and synthetic substances have been shown to activate the mitophagy process, thereby extending lifespan and promoting health in various model systems. The activity of some of these substances is mediated through conserved signaling pathways, including the insulin and antioxidant defense pathways.
- Developing cocktails containing mitophagy-activating compounds can potentially increase medical efficacy for treating age-related diseases and improving healthspan. Additionally, including substances that activate mitochondrial biogenesis could further amplify the impact of these formulations.

## Open Access

Open access for this article was enabled by the participation of Hebrew University of Jerusalem in an all-inclusive *Read & Publish* agreement with Portland Press and the Biochemical Society under a transformative agreement with MALMAD.

## Abbreviations

AMPK: AMP-activated protein kinase
BNIP3: BCL2 interacting protein 3
CaMK: Ca^2+^/calmodulin dependent kinase
FUNDC1: FUN 14 domain-containing 1
HIF-1: Hypoxia Inducible Factor 1
IMM: inner mitochondrial membrane
LIR: LC3 interacting region
MAC: mitophagy activating compound
NDP52: nuclear dot protein 52
OMM: outer mitochondrial membrane
PINK1: PTEN-induced kinase 1
ROS: reactive oxygen species
TBK1: TANK-binding kinase 1
TFs: transcription factors
VDAC1: voltage-dependent anion channel-1
